# Classification of Raw Stingless Bee Honeys by Bee Species Origins Using the NMR- and LC-MS-Based Metabolomics Approach

**DOI:** 10.3390/molecules23092160

**Published:** 2018-08-28

**Authors:** Muhammad Taufiq Atsifa Razali, Zaim Akmal Zainal, M. Maulidiani, Khozirah Shaari, Zulkifli Zamri, Mohd Zainuri Mohd Idrus, Alfi Khatib, Faridah Abas, Yee Soon Ling, Lim Leong Rui, Intan Safinar Ismail

**Affiliations:** 1 Laboratory of Natural Products, Institute of Bioscience, Universiti Putra Malaysia, 43400 Serdang, Selangor, Malaysia; atsifa79@gmail.com (M.T.A.R.); miaz1501@gmail.com (Z.A.Z.); maulidiani@upm.edu.my (M.M.); khozirah@yahoo.com.my (K.S.); faridah_abas@upm.edu.my (F.A.); 2 Syamille Agrofarm & Resort Sdn. Bhd., Lot 3749 & 3750, Jalan Lata Perahu, Kampung Chuar Hulu, Mukim Chegar Galah, 33020 Kati, Kuala Kangsar, Perak, Malaysia; zulkiflizamri@yahoo.com (Z.Z.); ctAteh18@yahoo.com (M.Z.M.I.); 3 Department of Pharmaceutical Chemistry, Kulliyyah of Pharmacy, International Islamic University Malaysia (IIUM), Jalan Sultan Ahmad Shah, Bandar Indera Mahkota, 25200 Kuantan, Pahang, Malaysia; alfikhatib@iium.edu.my; 4 Biotechnology Research Institute, Universiti Malaysia Sabah, Jalan UMS, 88400 Kota Kinabalu, Sabah, Malaysia; lingyeesoon@ums.edu.my (Y.S.L.); lrlim92@gmail.com (L.L.R.)

**Keywords:** classification, stingless bee honey, bee species origins, metabolomics, NMR, LC-MS, chemometrics

## Abstract

The official standard for quality control of honey is currently based on physicochemical properties. However, this method is time-consuming, cost intensive, and does not lead to information on the originality of honey. This study aims to classify raw stingless bee honeys by bee species origins as a potential classifier using the NMR-LCMS-based metabolomics approach. Raw stingless bee honeys were analysed and classified by bee species origins using proton nuclear magnetic resonance (^1^H-NMR) spectroscopy and an ultra-high performance liquid chromatography-quadrupole time of flight mass spectrometry (UHPLC-QTOF MS) in combination with chemometrics tools. The honey samples were able to be classified into three different groups based on the bee species origins of *Heterotrigona itama*, *Geniotrigona thoracica*, and *Tetrigona apicalis*. d-Fructofuranose (*H. itama* honey), β-d-Glucose, d-Xylose, α-d-Glucose (*G. thoracica* honey), and l-Lactic acid, Acetic acid, l-Alanine (*T. apicalis* honey) ident d-Fructofuranose identified via ^1^H-NMR data and the diagnostic ions of UHPLC-QTOF MS were characterized as the discriminant metabolites or putative chemical markers. It could be suggested that the quality of honey in terms of originality and purity can be rapidly determined using the classification technique by bee species origins via the ^1^H-NMR- and UHPLC-QTOF MS-based metabolomics approach.

## 1. Introduction

Meliponiculture (beekeeping with stingless bees) is a well-known tradition in tropical countries. Nowadays, meliponiculture is much more practiced than beekeeping with honey bees of the genus *Apis* (apiculture) for honey production. As stipulated in the Codex Alimentarius Commission [1], honey is defined as:

The natural sweet substance produced by honey bees from the nectar of plants or from secretions of living parts of plants or excretions of plant sucking insects on the living parts of plants, which the bees collect, transform by combining with specific substances of their own, deposit, dehydrate, store, and leave in the honey comb to ripen and mature. Blossom Honey or Nectar Honey is the honey which comes from nectars of plants. Honeydew Honey is the honey which comes mainly from excretions of plant sucking insects (Hemiptera) on the living parts of plants or secretions of living parts of plants.

This definition is specific to honey produced by honey bees, which could be any member of the genus *Apis*. However, the suggestion to expand the official definition of honey towards inclusion of the stingless bee has been systematically rejected for the last 10 years [2]. Therefore, the term divine elixir has been suggested for stingless bee honey as a possible alternative [3]. Although there is no recent revision made on the current definition of honey by Codex Alimentarius Commission, the term of stingless bee honey has still often been used instead of divine elixir.

The quality of honey is based on purity and originality. Purity of honey is determined by its physicochemical properties, whereas originality depends on several factors such as botanical, geographical, and entomological origins. At present, the production of stingless bee honey does not meet the great demand from consumers, thus leading to high prices and an increasing number of fraudulent practices in the market. Similar to *Apis* honeys, many problems have been encountered in terms of determining the purity and originality of stingless honey [4,5].

Adulteration and artificial honey are examples of problems related to purity of honey. An adulterated honey was produced when a small portion of pure honey is mixed with larger portion of high-fructose corn syrup, synthetic honey-flavouring agent or even with other types of pure honey. An example of this is the practice of diluting the high commercial value of unifloral honey (e.g., some New Zealand honeys (*L. scoparium* (manuka), *Kunzea ericoides* (kanuka), and *Trifolium* spp. (clover)) with those of lower commercial value honey (e.g., polyfloral honey) [4]. The artificial honey is absolutely made from sugar or corn syrups together with other additive ingredients. Whereas, mislabelling of floral or geographical origin and honey laundering are some examples related to originality of honey. Honey laundering involves the process where honey was filtered to remove pollen or soil that could be used to trace it back to its origin [5].

Several scientific methods have been used to determine the quality of honey. Sugar profiling based on chromatography [6,7], sensory analysis [8], and physicochemical analyses [2,9,10,11,12,13,14,15] are among the techniques used to determine the purity of honey.

The existing official standard for quality control of honey is based on the physicochemical characteristics of *Apis* honey such as acidity, ash, diastase activity, hydroxymethylfurfural (HMF), reducing sugars, sucrose, and water content. Nevertheless, the characteristics of stingless bee honey markedly differed from *Apis* honey in terms of viscosity, colour, and taste [16,17]. Therefore, Vit et al. [3] have proposed the quality standard for stingless bee honeys based on the accepted standard for *Apis mellifera*, which could be used to detect honey impurity in stingless bee honey.

However, these physicochemical analyses do not lead to any information about the botanical and geographical origin of honey [18]. Although artificial or adulterated honeys could be manipulated to conform to the quality standard of physicochemical parameters, the consistency of their metabolite composition could not be reproduced. Moreover, these physicochemical parameters are less appropriate to be used for monitoring the quality of stingless bee honey because of their low reproducibility, wherein often give a high variability in batch-to-batch results, tedious, time-consuming, and cost intensive.

This study is important because inquiry on whether bee species origin can be used as reliable classifier remains questionable. Nowadays, the perspective of a broader and lucrative market for natural and organic products indicates a need for more rapid and reliable method in quality control of honey. An untargeted metabolomics approach might be an improvement to the existing physicochemical approach in the detection of honey originality.

The untargeted metabolomics approach mainly consists of two interrelated steps; metabolite fingerprinting and metabolite profiling. Metabolite fingerprinting can be defined as high-throughput qualitative screening of metabolic composition of an organism or tissue with the primary aim of sample comparison and discrimination analysis. All steps from sample preparation, separation, and detection should be rapid and as simple as is feasible. Mostly, no attempt is initially made to identify the metabolites present. Metabolite fingerprinting is often used as a forerunner to metabolite profiling [19].

The next step is metabolite profiling of which can be defined as identification and quantification of the metabolites present in an organism. However, metabolite profiling is only feasible for a limited number of components, which are usually chosen on the basis of discriminant analysis or on molecular relationships based upon molecular pathways/networks [19].

It is possible to verify the botanical origin and exclude adulteration with sugars for honey [20]. Several metabolomics studies such as classification of honeys by different botanical origins [4,21,22,23,24,25,26,27] and geographical origins [23,28,29] have been carried out to address honey originality issues. To the extent of our knowledge, the untargeted metabolomics approach has not yet been applied in the classification of raw stingless bee honeys by different bee species origins within a specific geographical region.

There were also several reports from previous studies that used various types of predictors to determine the entomological origins of stingless bee honeys. For example, hexoses-to-maltose ratio in high performance liquid chromatography (HPLC) analysis was proposed as predictor (taxonomic marker) to predict entomological origins of stingless bee honeys [30]. In addition, Ramón-Sierra et al. [31] demonstrated the utility of protein profiles obtained from electrophoresis as predictor of entomological origins for stingless bee honeys. On the other hand, Kek et al. [32] showed that the entomological origins of stingless bee honeys (four different bee species) can be used as classifier to classify raw honeys using their chemical profiles and mineral contents. According to Ramón-Sierra et al. [31], it is possible to differentiate honey samples in terms of bee species origins using metabolomics tools. Therefore, this study aims to classify raw stingless bee honeys by bee species origins as a potential classifier using the NMR- and LC-MS-based metabolomics approach.

## 2. Materials and Methods

### 2.1. Species Identification

Voucher specimens of three stingless bee species identified were deposited at the Centre for Insect Systematics (CIS), Faculty of Science and Technology, Universiti Kebangsaan Malaysia, Bangi, Selangor with the following accession numbers: *Heterotrigona itama* (CIS-TRI-2014-002), *Geniotrigona thoracica* (CIS-TRI-2014-0001), and *Tetrigona apicalis* (CIS-TRI-2014-0004).

### 2.2. Collection of Honey Samples

Honey samples from the three stingless bee species were collected from the meliponiculture site of the Syamille Agrofarm and Resort (4.8992° N, 100.8964° E), Perak, Malaysia (Figure S1). The stingless bee hives were distributed in random spots within a land area of 48,562.3 m^2^ and were surrounded by several species of fruit trees (Myrtaceae, Meliaceae, Oxalidaceae, Moraceae, Rutaceae, Arecaceae), ornamental trees (Polygonaceae, Asteraceae, Rubiaceae), and resin-secreting trees (Dipterocarpaceae). These hives were made from naturally hollowed tree trunks, topped with wooden nest boxes, inside were cerumen pots of resins and wax mixtures to store nectar collected by the stingless bee [33]. For each species of stingless bee, honey samples were collected from 30 cerumen pots from six different nest boxes. Hence, five cerumen pots from each nest box. The collection was made between 7:00 a.m. and 11:30 a.m. All samples were kept in a cooler box with ice for transportation to the laboratory, prior to storing in the chiller at 4 °C until further analysis.

### 2.3. ^1^H-NMR Spectroscopy

#### 2.3.1. Chemicals and Reagents

Potassium dihydrogen phosphate (KH_2_PO_4_) and sodium deuteroxide solution (NaOD, 30% by weight in D_2_O) were purchased from Merck Sharp & Dohme (Kenilworth, NJ, USA). Methanol-d4 (CD_3_OD, 99.8%), deuterium oxide (D_2_O, 99.9%), and sodium-3-trimethylsilylpropionate (TMSP-2,2,3,3-d4, 98%) were purchased from Cambridge Isotope Laboratories, Inc. (Tewksbury, MA, USA).

#### 2.3.2. Sample Preparation

The chilled honey samples were allowed to sit at room temperature for at least 30 min prior preparation for data acquisition by ^1^H-NMR spectroscopy [34]. The phosphate buffer was prepared by dissolving 1.232 g of KH_2_PO_4_ and 10 mg of TMSP (0.01%) in 100 mL of D_2_O. The buffer solution was then adjusted to pH 6 with 1 M NaOD solution.

Honey samples for ^1^H-NMR measurement were prepared according to the procedure described by Kim et al. [34] with slight modifications. Honey in 5 mg was dissolved in 120 µL of deuterated methanol, followed by 480 µL of phosphate buffer. After centrifugation at 10,000 revolutions per min (RPM) for 2 min, 600 µL of the supernatant was pipetted into a round bottom NMR tube (4.97 mm × 4.2 mm, 178 mm) by Norell (Morganton, VA, USA) for data acquisition.

#### 2.3.3. Data Acquisition

The ^1^H-NMR spectra of stingless bee honey samples were acquired in duplicates, at 25 °C, on a 500 MHz Unity Inova NMR spectrometer (Varian Inc., California, CA, USA). Each spectrum was acquired over a spectral width of 0–10 ppm, using 64 scans and acquisition time of 256.8 s. The presaturation pulse sequence was used to suppress residual water signal with low power selective irradiation. D_2_O was used as internal lock and TMSP-2,2,3,3-d4 was used as reference standard at 0.00 ppm.

#### 2.3.4. Data Pre-processing

^1^H-NMR spectra were automatically phased using VnmrJ version 2.3 A (Varian Inc., Palo Alto, CA, USA) and further pre-processed with automatic baseline correction (spline) using Chenomx NMR Suite version 6.1 (Chenomx Inc., Edmonton, AB, Canada). The spectral intensities were scaled to TMSP (set to 0.0 ppm) and residual methanol (3.24–3.33 ppm) and water (4.68–4.88 ppm) signals were excluded. The remaining spectral regions were divided into 0.04 ppm bins, giving a total of 238 integrated regions (X-variables) per spectrum. The data were then converted to ASCII format and imported to Microsoft Excel 2010. A total of 79 spectra of ^1^H-NMR were acquired, binned, and used to develop the classification model.

#### 2.3.5. Data Analysis

The resulting Microsoft Excel file were imported into SIMCA version 14.1 (MKS Data Analytics Solutions, Umeå, Sweden) and pareto-scaled for multivariate data analysis (MVDA). Prior to classification of the honey samples by OPLS-DA model, principal component analysis (PCA) was performed to obtain an overview of the basic variation among the honey samples and to determine the presence of outliers. The robustness of OPLS-DA model was validated by means of cross-validation and response permutation test using 100 random permutations.

#### 2.3.6. Characterization of Discriminant Metabolites

Variables in the OPLS-DA model with both values of variable importance in projection of greater than one (VIP > 1) and error bar not exceeding zero were considered as discriminant metabolites. Characterization of discriminant metabolites was carried out based on the match between experimental ^1^H-NMR signals and reference compound of in-house library of Chenomx Profiler version 8.2 (Chenomx Inc., Edmonton, AB, Canada).

The identity of characterized metabolites was further supported by comparison of two-dimensional (2D) ^1^H-^1^H *J*-resolved and heteronuclear single quantum correlation (HSQC) experiments with online databases such as the Human Metabolome Database (HMDB), Kyoto Encyclopedia of Genes and Genomes (KEGG), PubChem, and ChemSpider.

### 2.4. UHPLC-QTOF Mass Spectrometry

#### 2.4.1. Chemicals and Reagents

LC-MS grade methanol (MeOH) and acetonitrile (ACN) were purchased from J.T. Baker (Philipshurg, MJ, USA). LC-MS grade ammonium acetate (NH_4_OAc) and formic acid (HCOOH) and sodium formate were purchased from Sigma-Aldrich (St. Louis, MO, USA). Sodium chloride (NaCl) was from Merck (Darmstadt, Germany). Water used in LC was purified using Milli-Q system (Millipore, Milford, MA, USA) at resistivity of >18.2 MΩ⋅cm.

#### 2.4.2. Sample Preparation

Stingless bee honey samples (in three biological replicates per species) were randomly selected from the collected 30 replicates (Section 2.2). An aliquot of 100 µL of each honey sample was mixed with 500 µL of MeOH:ACN:Water (1:1:1 *v*/*v*) and vortexed until fully mixed. The mixture was centrifuged at 9520 relative centrifugal force (RCF) at 4 °C for 30 min. The supernatant was then filtered and transferred into sample vial and stored at −80 °C until further analysis. For quality control (QC) purpose, all the extracted samples (*n* = 9) were mixed.

#### 2.4.3. Data Acquisition

The extracted samples were subjected to Vanquish^TM^ Horizon UHPLC system (Thermo Fisher Scientific, Waltham, MA, USA) coupled with electrospray ionisation Impact II QTOF-mass spectrometry system (Bruker Daltonics, Bremen, Germany). Honey sample of 10 µL was injected into Kinetex F5 LC column (2.1 mm × 100 mm, 2.6 μm; Phenomenex, Torrance, CA, USA). The column was maintained at 40 °C and eluted at a flow rate of 600 μL/min during analysis. The mobile phase was composed of solvent A (mixture of H_2_O, 0.1% HCOOH, and 1% NH_4_OAc (10 mM)) and solvent B (mixture of acetonitrile/methanol [6:4 *v*/*v*], 1% of 0.1% HCOOH and 1% NH_4_OAc (10 mM)). The gradient elution program was initiated from 1% to 40% solvent B in 5 min, followed by 100% solvent B from 5.1 min to 8 min and maintained for the next 2 min. The column was conditioned with the initial gradient for 3 min before each sample injection.

The mass data acquisition was set to *m*/*z* values of 50–1500 amu. Positive and negative mode of electrospray ionisation (ESI) were deployed at 4200 V and −4200 V, respectively. The ion source conditions were set as follows: gas temperature of 300 °C, drying gas flow at 12 L/min, and nebulizer flow at 5.0 bar. Mass calibration standard of sodium formate (10 mM) was introduced post-column via a 6-port valve at 0.1–0.3 min. The *m*/*z* values of acquired data were calibrated against the introduced sodium formate and subsequently converted into netCDFdata format (*.cdf) using ACD/Spectrus Processor version 2017.1.3.

#### 2.4.4. Data Processing

The mass netCDF files were processed using MZmine version 2 [35] to compensate for variations in the retention times and *m*/*z* values between each analysis. Upon completion of the data processing, the mass spectral data were exported into a file with a comma-separated values data format (*.csv) as a peak list table, with rows representing the honey samples and columns representing the integrated and normalized peak areas (*m*/*z* values and retention times).

#### 2.4.5. Data Analysis

The generated csv file (*.csv) was imported to SIMCA version 14.1 (MKS Data Analytics Solutions, Umeå, Sweden) and set with unit variance (UV) scaling. As with the NMR data, PCA overview was carried out prior to the PLS-DA model. The validity of the PLS-DA model was also verified by cross-validation and response permutation tests of 100 random permutations.

#### 2.4.6. Characterization of Diagnostic Ions

ESI ionizes polar compounds more efficiently with basic sites (ESI^+^) or acidic sites (ESI^−^) [36]. The *m*/*z* values of precursor ions (ESI^+^ and ESI^−^) in the PLS-DA model with VIP > 1 and with error bar not exceeding zero were considered as diagnostic ions. Characterization of diagnostic ions could be performed based on comparison with online databases such as Metlin, KEGG, PubChem, ChemSpider, and MetFrag. Fragmentation of diagnostic ions was also generated for essential uses in structural elucidation and confirmation of the metabolite identity.

## 3. Results

### 3.1. An Overview by PCA

#### 3.1.1. PCA of ^1^H-NMR Spectral Data

An overview of PCA on ^1^H-NMR metabolite fingerprints was performed to identify outliers in the data using Hotelling’s T2. The PCA score plot (R2X = 0.991; Q2 = 0.946), showed no observations outside the tolerance ellipse based on Hotelling’s T2, indicating that there were no strong outliers present in the data (Figure S2). The three types of honey samples were seen to be separated into two main clusters by PC2. Although the *H. itama* honey samples were separated from those of *T. apicalis*, there was partial overlap of the *G. thoracica* honey samples with those of both *H. itama* and *G. thoracica*. Therefore, a discriminant-based classification model was developed to classify the honey samples.

#### 3.1.2. PCA of UHPLC-QTOF Mass Spectrometric Data

The PCA score plots were constructed using positive (ESI^+^) (Figure S3) and negative (ESI^−^) precursor ions (Figure S4). Both score plots (ESI^+^: R2X = 0.61; Q2 = 0.253, ESI^−^: R2X = 0.901; Q2 = 0.799) showed no strong outliers in the mass data of honey samples. The honey samples of three species of stingless bee were clustered well into three separate groups, justifying further data analysis using the PLS-DA classification model.

### 3.2. Classification Models of MVDA

#### 3.2.1. OPLS-DA (^1^H-NMR Spectral Data)

**Figure 1:**
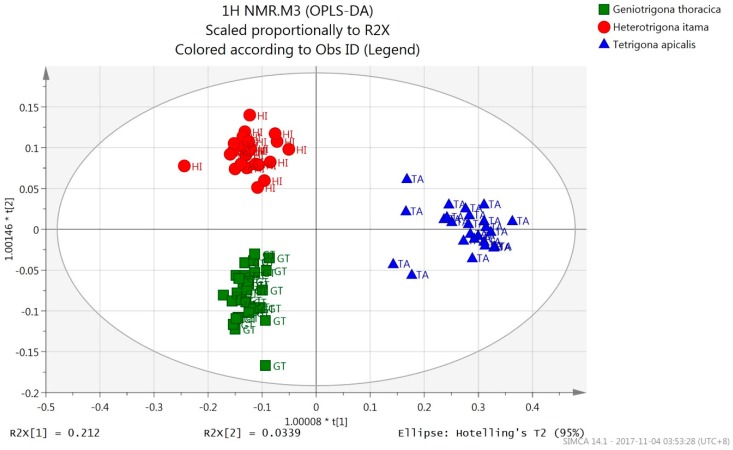
The OPLS-DA score scatter plot of ^1^H-NMR spectral data.

An OPLS-DA model was built using the ^1^H-NMR data of the honey samples. As shown by the OPLS-DA score plot (Figure 1), the honey samples were classified into three separate groups with 100% correct classification (Table 1) according to their bee species origins. Honey samples from *T. apicalis* were discriminated from those of *H. itama* and *G. thoracica* honey by PC1, while *H. itama* honey samples were discriminated from those of *G. thoracica* by PC2.

For each component in PLS and OPLS models and their corresponding DA-extensions, summary of fit plot displays R2Y and Q2 bars. R2Y is the percent of variation of the training set-Y with OPLS-explained by the Y-predictive components. R2Y is a measure of fit, i.e., how well the model fits the data wherein a poor R2Y value is given when there is poor reproducibility (noisy data) in the training data set, or when for other reasons X does not explain Y. Q2 indicates how well a model could predict a new data set. Q2 > 0.5 indicates good predictability and Q2 > 0.9 is excellent. The difference between R2Y and Q2 larger than 0.2–0.3 indicates the presence of a few outlying data points [37]. A poor Q2 value is given when the data is noisy, or when the relationship between X to Y is poor, or when the model is dominated by a few scattered outliers. In this study, the OPLS-DA model demonstrated an optimum goodness of fit (R2Y = 0.921) and satisfied the criteria for good predictability (Q2 = 0.838), and the difference between R2Y and Q2 was 0.083.

Response permutation testing aims to assess the risk whether the OPLS-DA model is spurious or not. A model could fit well to the training set, however it might not predict Y well for the new observations. R2Y and Q2 of the original model are compared with R2Y and Q2 of several models based on data whereby the order of the Y-observations has been randomly permuted, while the X-matrix was kept constant. The model is validated when R2Y-intercept does not exceed 0.3–0.4 and that the Q2-intercept does not exceed 0.05 (Eriksson et al., 2006). The present results gave Y intercepts of 0.382 (R2), −0.58 (Q2) for *H. itama* honey; 0.373 (R2) and −0.604 (Q2) for *G. thoracica* honey and, 0.364 (R2) and −0.641 (Q2) for *T. apicalis* honey (Figure S5). Hence, the OPLS-DA model was verified as valid.

#### 3.2.2. PLS-DA (UHPLC-QTOF Mass Spectrometric Data)

PLS-DA classification model was used instead of OPLS-DA model for UHPLC-QTOF mass spectral data. This is due to the response permutation tests of OPLS-DA models for *m*/*z* values of both ESI^+^ (Figure S6) and ESI^-^ precursor ions (Figure S7) were shown to be overfitting.

**Figure 2:**
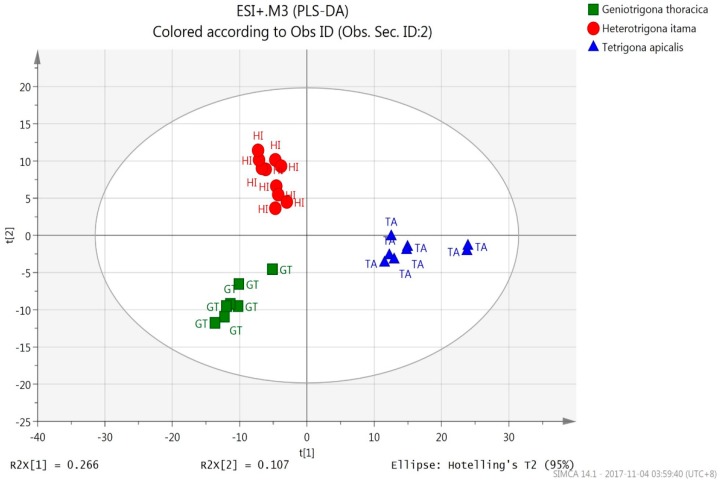
PLS-DA score scatter plot for *m*/*z* values of ESI^+^ precursor ions by UHPLC-QTOF MS data.

**Figure 3:**
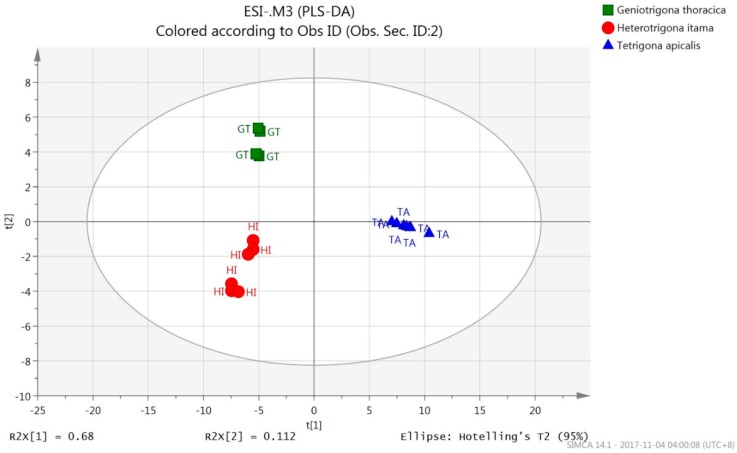
PLS-DA score scatter plot of for *m*/*z* values of ESI^−^ precursor ions by UHPLC-QTOF MS data.

The PLS-DA score plots for *m*/*z* values of both ESI^+^ (Figure 2) and ESI^−^ precursor ions (Figure 3) for the honey samples were clearly classified into three discernible groups with 100% correct classification (Table 2) according to their bee species origins. *T. apicalis* group was discriminated from that of *H. itama* and *G. thoracica* by PC1, while *H. itama* group was discriminated from *G. thoracica* by PC2. Both PLS-DA models (ESI^+^: R2Y = 0.922, Q2 = 0.833, R2Y − Q2 = 0.089; ESI^−^: R2Y = 0.989, Q2 = 0.976, R2Y − Q2 = 0.013) demonstrated the optimum goodness of fit and satisfied the criteria for good predictability.

It is very crucial to validate the PLS-DA model via response permutation testing because there is a risk of data overfitting in this supervised classification model. Response permutation testing for the PLS-DA model of ESI^+^ precursor ions for all honeys showed Y-intercepts of 0.391 (R2) and −0.233 (Q2) for *H. itama*; 0.364 (R2) and −0.234 (Q2) for *G. thoracica*, and 0.31 (R2), −0.3 (Q2) for *T. apicalis* (Figure S8). In addition, the PLS-DA model of ESI^-^ precursor ions of *H. itama* gave Y-intercepts of 0.339 (R2) and −0.369 (Q2); *G. thoracica* of 0.316 (R2) and −0.365 (Q2), and *T. apicalis* of 0.339 (R2) and −0.341 (Q2) (Figure S9). Hence, all of these PLS-DA classification models for both precursor ions were verified valid.

Based on the results of OPLS-DA and PLS-DA classification models, it was obvious that the honey samples were able to be classified into three different groups by bee species origins. The metabolite contents of honey differ according to bee species origins because stingless bees might have selective affinity towards certain types of floral nectars. There are a few types of flowers that stingless bees do not frequent, typically flowers with long corolla and have very little nectar or low brix value. However, stingless bees have an affinity for inflorescences, flowers with long anters, short corollas, and clustered flower head [38]. In addition, the metabolites contents are varied due to different stingless bee species might secrete different types of enzymes to the foraged nectar. According to Kek et al. [32], the composition of honey is probably affected by the type of bee because honey-making process is highly related to enzymes added by the bees. Therefore, bee species origins could be suggested as reliable classifier in rapid determination of honey quality in terms of originality and purity using NMR-LCMS-based metabolomics approach.

### 3.3. Metabolite Identification

#### 3.3.1. Characterization of Discriminant Metabolites (^1^H-NMR Spectral Data)

The loading column plots were generated to display the interclass separation based on binned regions. Variables with VIP > 1 (Figure S10) and with error bar not exceeding zero (Figure S11a–c) were designated as discriminant metabolites. L-Lactic acid was determined as the most influential metabolite in VIP plot of OPLS-DA model for ^1^H-NMR spectral data.

**Figure 4:**
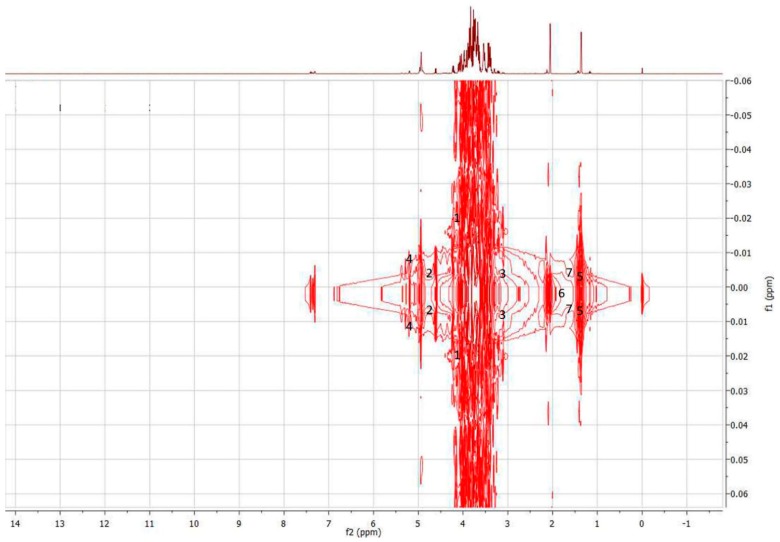
^1^H-^1^H *J*-resolved spectrum of *T. apicalis* honey in the region of 0.3–10.0 ppm. **1**, d-Fructofuranose; **2**, β-d-Glucose; **3**, d-Xylose; **4**, α-d-Glucose; **5**, l-Lactic acid; **6**, Acetic acid; **7**, l-Alanine.

The discriminant metabolites listed in Table 3 were proposed as chemical markers for each honey type. d-Fructofuranose was the major discriminant for *H. itama* honey (Figure S12), while β-d-Glucose, and d-Xylose, and α-d-Glucose were the discriminant metabolites for *G. thoracica* honey (Figure S12). On the other hand, the variables contributing most significantly to discrimination of *T. apicalis* honey from both *H. itama* and *G. thoracica* honey were l-Lactic acid, Acetic acid, and l-Alanine (Figure S12). The identities of all discriminants were further verified based on the results obtained from ^1^H-^1^H *J*-resolved experiments (Figure 4 and Figure S12).

#### 3.3.2. Characterization of Diagnostic Ions (UHPLC-QTOF Mass Spectrometric Data)

The diagnostic ions of ESI-MS fingerprints responsible for group separation among *H. itama*, *G. thoracica*, and *T. apicalis* honeys were as listed in Table 4a–c, respectively. There were uncharacterized metabolites that differentiated the three honey types with *m*/*z* value of 446.203 [M + H]^+^ being the most influential diagnostic ion (VIP = 1.91) in PLS-DA model of UHPLC-QTOF mass spectrometric data (Table 4a). The major contributor to *H. itama* honey was 446.203 *m*/*z* [M + H]^+^ (Table 4a), while 401.311 *m*/*z* [M − H]^−^ was the diagnostic ion for *G. thoracica* honey (VIP = 1.83) (Table 4b). *T. apicalis* honey was discriminated from *H. itama* and *G. thoracica* honey by *m*/*z* of 277.178 [M + H]^+^ with VIP = 1.45 (Table 4c).

VIP value of more than one (VIP > 1), which has an above average influence on Y summarised the influence of every term in the matrix X on all the Y’s [37]. The variability in the metabolite fingerprints of stingless bee honey related to the bee species origins observed via ^1^H-NMR spectroscopy (Q2 = 0.838) and UHPLC-QTOF mass spectrometry (ESI^+^: Q2 = 0.833; ESI^−^: Q2 = 0.976) can function as good predictors to determine bee species origins of stingless bee honey. An accurate prediction of bee species origins by such good predictors i.e., discriminant metabolites (^1^H-NMR spectral data) and diagnostic ions (UHPLC-QTOF mass spectrometric data) for the determination of honey originality has been established.

## 4. Discussion and Conclusions

d-Fructofuranose was described as a discriminant metabolite for *H. itama* honey, whereas β-d-Glucose, d-Xylose, and α-d-Glucose were responsible for the discrimination of *G. thoracica* honey (Table 3). The hydrolysis of sucrose from nectar into d-Glucose and d-Fructofuranose is mainly catalysed by invertase enzymes (sucrase, glucosidase, transglucosidase), which are secreted by cephalic glands of stingless bee workers [39,40]. d-Xylose was also found in the honey as reported by Ohmenhaeuser et al. [25].

Acetic and l-Lactic acids of *T. apicalis* honey (Table 3) are the products of fermentation from carbohydrates. Basically, there are three main categories of fermentation in stingless bee honey: Alcoholic, acetic, and lactic fermentation. The alcoholic fermentation (indicated by bubbles and foam) is performed by yeasts, which convert carbohydrates (sugar) into alcohol and CO_2_. Subsequently, acetic fermentation is performed under aerobic conditions by certain strains of bacteria (commonly Bacillus), which convert alcohol molecules and O_2_ into acetic acid and water. In addition, lactic fermentation can occur when bacteria mostly convert carbohydrates into lactic acid and water or other organic molecules although yeasts and other fungi can perform similar function. However, those three categories of fermentation can naturally be mixed in stingless bee honey via enzymatic reaction of those microorganisms [40]. Another discriminant metabolite of *T. apicalis* was L-Alanine (Table 3), which also found in the honey as reported by Boffo et al. [41].

QTOF MS-based identification of metabolites may consist of three main steps, i.e., characterization of metabolites guided by mass spectral database, validation of metabolite structure, and confirmation of metabolite identity (targeted metabolite profiling) [42]. In this study, diagnostic RT, *m*/*z* values of precursor ions (ESI^+^ and ESI^−^), and MS-MS fragment ions as well as their corresponding intensities of detected ions represent as distinct ESI-MS fingerprints for each type of stingless bee honey. However, characterization of all diagnostic ions could not be completed *m*/*z* values of precursor ions could not be matched with MS-MS fragment ions in any databases.

The present study demonstrates the feasibility of using untargeted metabolomics approach for rapid classification of raw stingless bee honeys by bee species origins. Two complementary approaches, ^1^H-NMR and UHPLC-QTOF MS are more appropriate for honey quality control. ^1^H-NMR spectroscopy provides rapid detection by minimum sample preparation, non-destruction of the samples, and high reproducibility of data. These advantages are complemented by the UHPLC-QTOF MS, which enables high sensitivity or trace-level detection of metabolites with only minimal amounts of sample. LCMS also provides the possibility to extend the range of compounds detected by using different ion sources (e.g., electrospray ionization or atmospheric chemical ionization) and/or ion modes (positive and negative). The variability of metabolites detected exhibits the complementary nature of ^1^H-NMR spectroscopy and UHPLC-QTOF MS techniques (Table 3 and Table 4(a–c)).

Nevertheless, the numbers of bee species origins involved in the present study were limited to only three stingless bee species. Further research need to be conducted on many other species of stingless bee, as mentioned by Schwarz [43] and Rasmussen [44] in more diversified geographical coverage of beekeeping areas.

In conclusion, this study showed that stingless bee honeys can be classified by bee species origin (as reliable classifier) using a robust untargeted metabolomics approach to determine the honey originality. As a result, this study may give a significant impact to the global meliponiculture and apiculture industries in improving the efficiency of honey quality control.

## Figures and Tables

**Table 1:** The misclassification table for OPLS-DA model of three types of stingless bee honey.

Species Origin	% Correct	Classes of Honey (OPLS-DA)		
		*H. itama*	*G. thoracica*	*T. apicalis*
*H. itama*	100	25	0	0
*G. thoracica*	100	0	29	0
*T. apicalis*	100	0	0	25
**Total**		25	29	25
**Average**	100			

**Table 2:** The misclassification table for PLS-DA model of *m*/*z* values of ESI^+^ and ESI^-^ precursor ions.

Species Origin	% Correct	Classes of Honey (ESI^+^)			Classes of Honey (ESI^-^)		
		*H. itama*	*G. thoracica*	*T. apicalis*	*H. itama*	*G. thoracica*	*T. apicalis*
*H. itama*	100	10	0	0	6	0	0
*G. thoracica*	100	0	7	0	0	4	0
*T. apicalis*	100	0	0	8	0	0	7
**Total**		10	7	8	6	4	7
**Average**	100						

**Table 3:** List of tentative discriminant metabolites (chemical markers) based on ^1^H-NMR spectral data for each types of stingless bee honey.

VIP > 1	Binned Region	^1^H-NMR Characteristics Signals	Online HMDB (^1^H-NMR)	*J*-Resolved	HSQC (^1^H-^13^C) Characteristics Signals	Online HMDB (HSQC, ^1^H-^13^C)	Tentative Discriminant Metabolites	Honey Types
4.45	3.68	3.68 (m)					d-Fructofuranose	*H. itama*
3.56	3.52	3.52 (m)						
3.32	4.00	4.00 (m)						
3.13	4.08	4.10 (d, 8.5)	4.118 (m)	d	4.1 (79.747)	4.1055 (78.2044)		
2.62	3.76	3.76 (m)						
2.09	3.96	3.96 (m)						
2.19	4.60	4.61 (d, 7.9)	4.634 (d, 7.957)	d	4.61 (94.048)	4.6333 (98.7123)	β-d-Glucose	*G. thoracica*
1.77	3.20	3.21 (dd, 9.4, 8.71)	3.21 (dd, 9.33)	3.21 (dd, 7.90)	d	3.2140 (76.4140)	3.2144 (76.9117)		d-Xylose
1.44	5.20	5.20 (d, 3.7)	5.223 (d, 3.677)	d	5.2 (93.293)	5.2241 (94.9364)	α-d-Glucose	
1.25	2.00	**2.05 (s)**	-	-	-	-	**Unassigned (2.05)**	
3.81	1.32	1.36 (d, 6.90)	1.32 (d, 6.96)	d	1.387 (21.841)	1.3142 (22.9033)	l-Lactic acid	*T. apicalis*
2.88	1.92	1.92 (s)	1.91 (s)	s	1.943 (26.751)	1.9059 (26.0899)	Acetic acid	
1.47	5.28	**5.28 (t, 3.6)**	**-**	-	-	-	**Unassigned (5.28)**	
1.22	1.44	1.43 (d, 7.00)	1.46 (d, 7.14)	d	1.450 (19.702)	1.4903 (19.0295)	l-Alanine	

**Table 4:** (a) List of diagnostic ions for *H. itama* honey based on UHPLC-QTOF mass spectrometric data. (b) List of diagnostic ions for *G. thoracica* honey based on UHPLC-QTOF mass spectrometric data. (c) List of diagnostic ions for *T. apicalis* honey based on UHPLC-QTOF mass spectrometric data.

**(a)**					
**VIP > 1**	**Var ID (Primary)**	**Ion**	**RT (min)**	**Experimental Precursor Ions (*m*/*z*)**	**Experimental MS-MS Fragment Ions (*m*/*z*)**
1.66	492	[M + H]^+^	1.44	193.087	165.092, 162.068, 147.044, 135.044, 133.065, 105.071
1.62	493	[M + H]^+^	1.50	151.076	
1.91	428	[M + H]^+^	1.55	446.203	
1.66	399	[M + H]^+^	1.55	105.070	
1.83	359	[M + H]^+^	1.56	122.096	106.073, 105.070, 103.054
1.79	396	[M + H]^+^	1.57	266.139	
1.78	397	[M + H]^+^	1.57	284.150	268.145, 267.143, 266.139, 249.132, 248.129, 164.107, 134.097, 105.070
1.77	451	[M + H]^+^	1.57	267.142	
1.72	431	[M + H]^+^	1.80	392.133	
1.71	401	[M + H]^+^	1.80	225.110	181.084, 165.055, 139.076, 121.065
1.15	490	[M + H]^+^	1.80	234.150	191.105, 189.091, 122.032, 121.029, 114.128
1.08	276	[M + H]^+^	3.03	362.327	
1.07	120	[M + H]^+^	4.94	310.311	
1.03	188	[M + H]^+^	4.95	695.361	
1.08	226	[M + H]^+^	5.04	637.307	
1.03	216	[M + H]^+^	5.04	695.360	659.294, 581.245, 359.032, 330.992, 289.006, 135.004
1.47	416	[M + H]^+^	5.13	358.309	178.945, 177.013, 136.006, 135.003, 132.987, 123.117, 120.987, 105.068, 104.992
1.22	418	[M + H]^+^	5.13	336.327	
1.05	470	[M + H]^+^	5.27	371.102	
1.57	19	[M + H]^+^	5.54	360.324	358.365, 135.004
1.56	124	[M + H]^+^	5.54	321.316	
1.55	8	[M + H]^+^	5.54	338.343	
1.52	107	[M + H]^+^	5.54	675.678	338.343, 321.316, 303.305, 149.133, 135.117, 111.117, 97.102
1.05	444	[M + H]^+^	6.07	679.366	
1.45	410	[M + H]^+^	6.09	366.374	
1.15	78	[M − H]^-^	1.00	668.224	
1.17	50	[M − H]^-^	1.69	495.183	
1.63	60	[M − H]^-^	1.73	493.168	
1.57	39	[M − H]^-^	1.82	119.114	
1.66	42	[M − H]^-^	1.83	353.112	227.051, 211.030, 190.984, 166.013, 165.009, 147.014, 120.048, 119.046
1.27	31	[M − H]^-^	1.83	147.098	
1.16	29	[M − H]^-^	1.83	165.102	
1.41	46	[M − H]^-^	1.86	206.118	
1.15	45	[M − H]^-^	2.37	201.075	
**(b)**					
**VIP > 1**	**Var ID (Primary)**	**Ion**	**RT (min)**	**Experimental Precursor Ions (*m*/*z*)**	**Experimental MS-MS Fragment Ions (*m*/*z*)**
1.62	126	[M + H]^+^	1.03	365.106	
1.32	475	[M + H]^+^	1.13	174.149	
1.32	283	[M + H]^+^	1.30	365.106	
1.44	301	[M + H]^+^	1.41	203.053	
1.33	348	[M + H]^+^	1.69	365.106	
1.44	521	[M + H]^+^	1.81	351.142	
1.46	502	[M + H]^+^	1.82	317.114	
1.46	502	[M + H]^+^	1.82	317.114	
1.33	501	[M + H]^+^	1.97	227.083	210.074, 209.071, 199.087, 181.076, 154.065
1.36	517	[M + H]^+^	1.99	521.272	519.256, 518.236, 517.228, 366.109, 365.106, 285.887, 218.941, 203.050, 185.042, 140.070, 135.004, 132.985
1.46	528	[M + H]^+^	2.01	301.118	
1.46	528	[M + H]^+^	2.01	301.118	
1.46	528	[M + H]^+^	2.01	301.118	
1.46	528	[M + H]^+^	2.01	301.118	
1.27	479	[M + H]^+^	2.05	321.131	319.210, 319.161, 281.016, 279.020, 187.060, 142.948
1.30	388	[M + H]^+^	2.08	183.091	182.154, 155.047, 127.016, 98.984
1.35	516	[M + H]^+^	2.11	551.283	
1.41	483	[M + H]^+^	4.91	439.375	
1.43	421	[M + H]^+^	5.27	367.319	
1.51	503	[M + H]^+^	5.35	393.334	
1.80	478	[M + H]^+^	5.49	467.408	
1.25	520	[M + H]^+^	5.73	481.387	
1.00	486	[M + H]^+^	5.78	637.469	
1.46	519	[M + H]^+^	5.87	391.320	
1.68	87	[M − H]^-^	4.86	345.255	
1.80	86	[M − H]^-^	5.42	373.283	
1.83	88	[M − H]^-^	5.93	401.311	
**(c)**					
**VIP > 1**	**Var ID (Primary)**	**Ion**	**RT (min)**	**Experimental Precursor Ions (*m*/*z*)**	**Experimental MS-MS Fragment Ions (*m*/*z*)**
1.07	364	[M + H]^+^	1.45	492.207	408.165, 332.243, 292.119, 264.124, 244.097, 166.086, 121.084, 120.081
1.13	183	[M + H]^+^	1.61	158.082	
1.12	245	[M + H]^+^	1.62	178.086	
1.22	110	[M + H]^+^	1.64	389.178	
1.23	171	[M + H]^+^	1.67	515.173	
1.26	250	[M + H]^+^	1.68	238.108	
1.11	208	[M + H]^+^	1.68	535.236	
1.27	362	[M + H]^+^	1.69	311.113	
1.27	111	[M + H]^+^	1.69	227.126	
1.30	140	[M + H]^+^	1.70	353.121	
1.19	146	[M + H]^+^	1.71	373.183	
1.21	119	[M + H]^+^	1.73	211.131	
1.14	137	[M + H]^+^	1.83	260.090	
1.04	191	[M + H]^+^	1.83	401.171	
1.02	373	[M + H]^+^	1.78	107.085	
1.00	365	[M + H]^+^	1.80	180.102	
1.07	369	[M + H]^+^	1.81	151.112	
1.19	85	[M + H]^+^	1.84	120.081	
1.15	142	[M + H]^+^	1.85	649.269	
1.30	141	[M + H]^+^	1.86	192.103	
1.02	195	[M + H]^+^	1.86	230.080	
1.17	215	[M + H]^+^	1.93	162.091	
1.09	381	[M + H]^+^	1.95	644.313	
1.17	382	[M + H]^+^	1.96	283.152	
1.17	382	[M + H]^+^	1.96	283.152	
1.15	246	[M + H]^+^	1.96	153.127	
1.15	246	[M + H]^+^	1.96	153.127	
1.28	17	[M + H]^+^	2.00	487.215	
1.26	69	[M + H]^+^	2.00	482.260	355.174, 335.095, 154.131, 153.128, 135.117, 115.039, 97.028
1.08	249	[M + H]^+^	2.00	171.138	
1.13	112	[M + H]^+^	2.02	153.127	
1.04	42	[M + H]^+^	2.04	355.173	
1.01	368	[M + H]^+^	2.14	253.142	
1.39	175	[M + H]^+^	2.23	293.173	
1.14	394	[M + H]^+^	2.26	307.152	
1.00	372	[M + H]^+^	2.26	267.158	
1.34	392	[M + H]^+^	2.30	195.138	
1.34	392	[M + H]^+^	2.30	195.138	
1.37	383	[M + H]^+^	2.33	249.146	
1.32	391	[M + H]^+^	2.38	253.179	
1.27	237	[M + H]^+^	2.38	291.157	
1.38	200	[M + H]^+^	2.41	439.230	
1.30	377	[M + H]^+^	2.42	221.154	
1.44	393	[M + H]^+^	2.44	217.159	
1.34	189	[M + H]^+^	2.45	235.170	
1.37	193	[M + H]^+^	2.47	423.235	
1.06	253	[M + H]^+^	2.47	251.165	
1.36	201	[M + H]^+^	2.48	275.162	
1.02	260	[M + H]^+^	2.49	233.154	
1.00	232	[M + H]^+^	2.52	421.219	
1.45	65	[M + H]^+^	2.61	277.178	
1.11	154	[M + H]^+^	2.63	223.169	
1.42	95	[M + H]^+^	2.73	219.175	
1.06	248	[M + H]^+^	2.76	237.185	
1.09	255	[M + H]^+^	2.77	261.183	
1.40	92	[M + H]^+^	2.89	511.340	
1.11	330	[M + H]^+^	3.05	272.259	
1.17	412	[M + H]^+^	3.67	359.030	
1.26	93	[M + H]^+^	3.73	359.030	
1.09	75	[M + H]^+^	4.06	711.131	
1.00	367	[M + H]^+^	4.95	359.030	358.368, 358.309, 342.310, 341.307, 285.279, 267.271, 136.007, 135.003, 123.117, 109.102
1.07	194	[M + H]^+^	5.00	359.030	
1.04	61	[M + H]^+^	5.45	983.202	
-	-	[M − H]^-^	-	-	-

## Supplementary Materials

The supplementary materials are available online.
