# Supplementary figures and images for: Classification of Raw Stingless Bee Honeys by Bee Species Origins Using the NMR- and LC-MS-Based Metabolomics Approach
Source: Molecules. 2018 Aug 28;23(9):2160. doi: 10.3390/molecules23092160 (PMC6225217; doi:10.3390/molecules23092160)

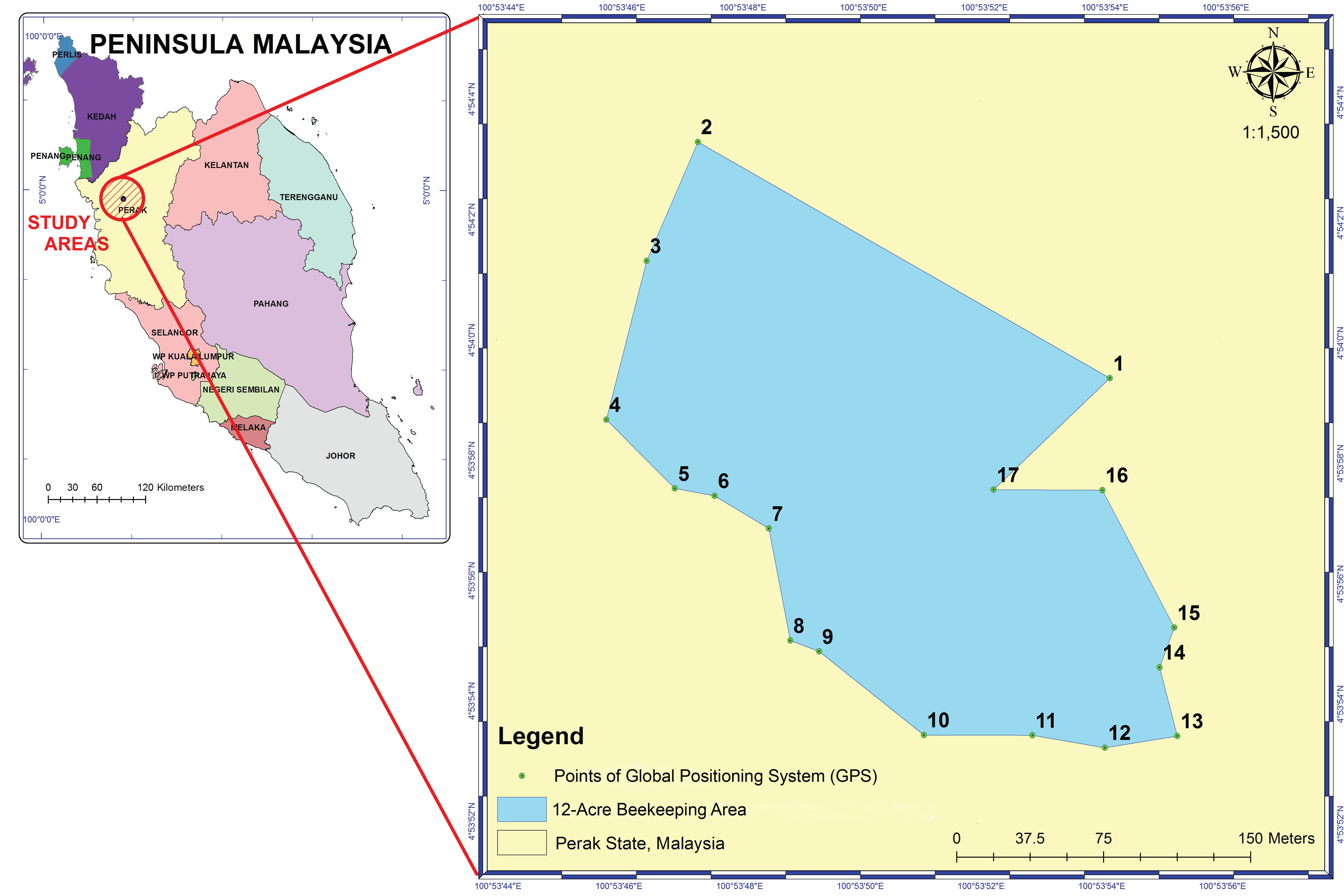

Supplement: Supplementary file 1 [file molecules-23-02160-s001.zip › Supplementary/Fig. 1-6c/Figure S1.tif]

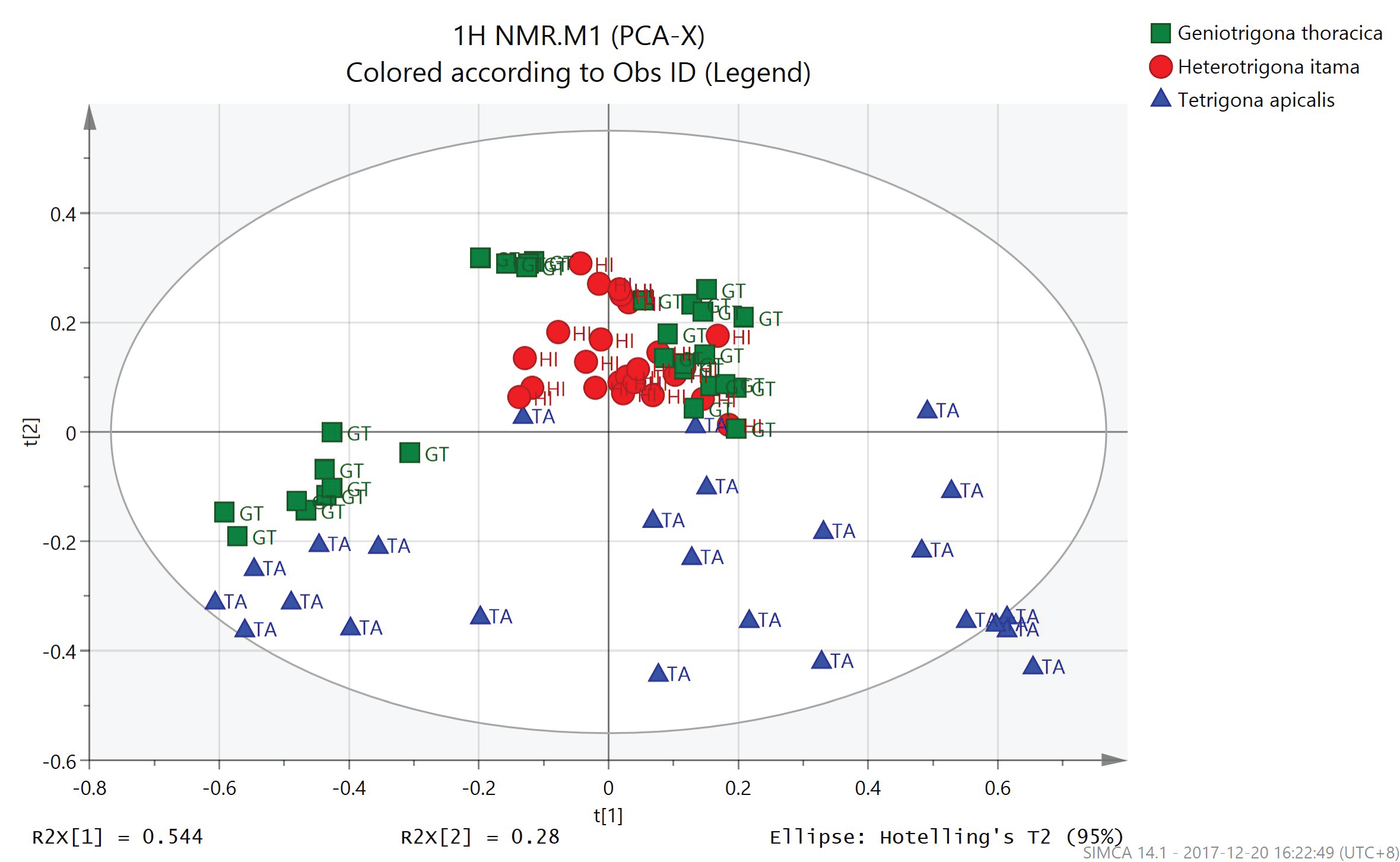

Supplement: Supplementary file 1 [file molecules-23-02160-s001.zip › Supplementary/Fig. 1-6c/Figure S2.tif]

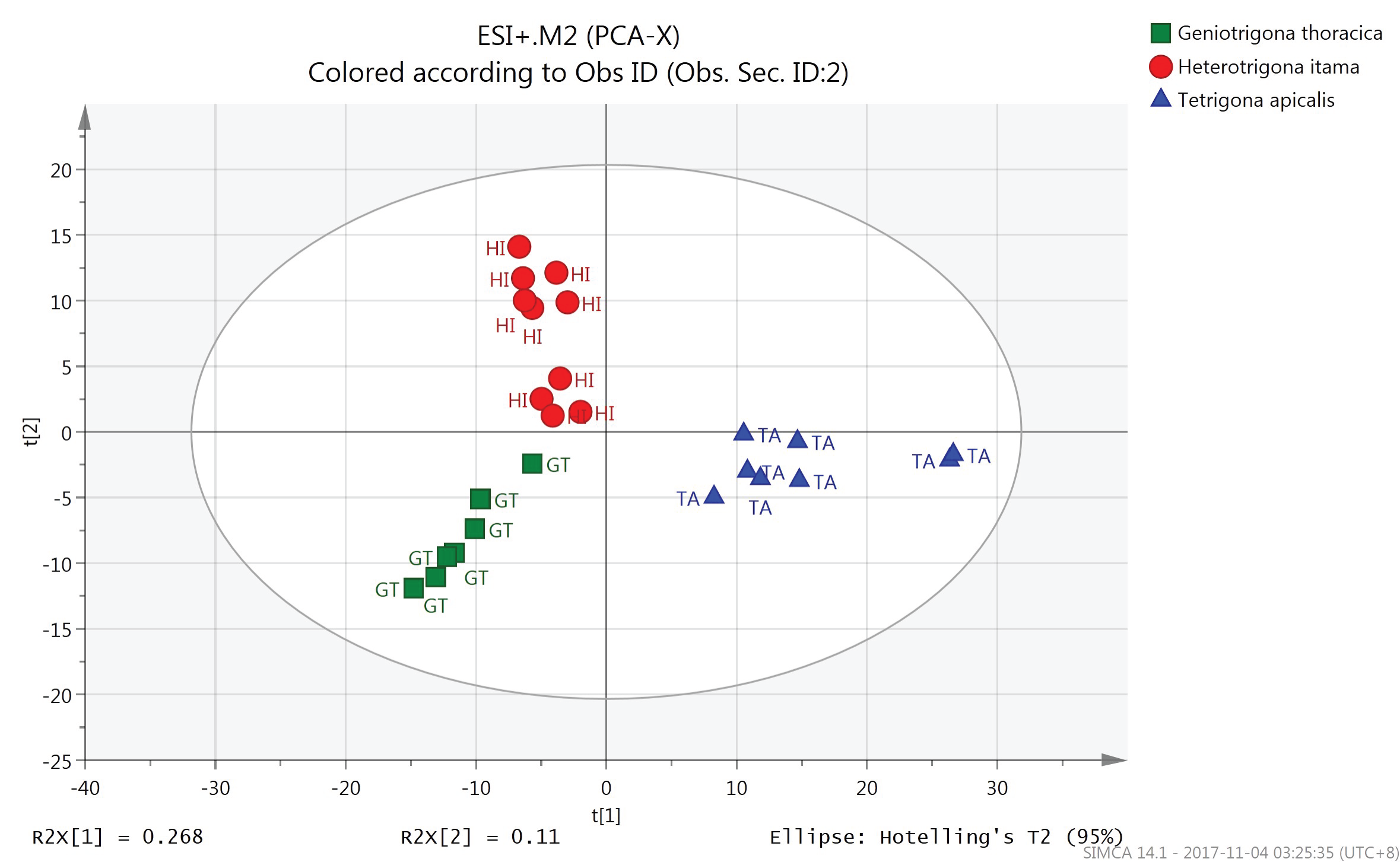

Supplement: Supplementary file 1 [file molecules-23-02160-s001.zip › Supplementary/Fig. 1-6c/Figure S3.tif]

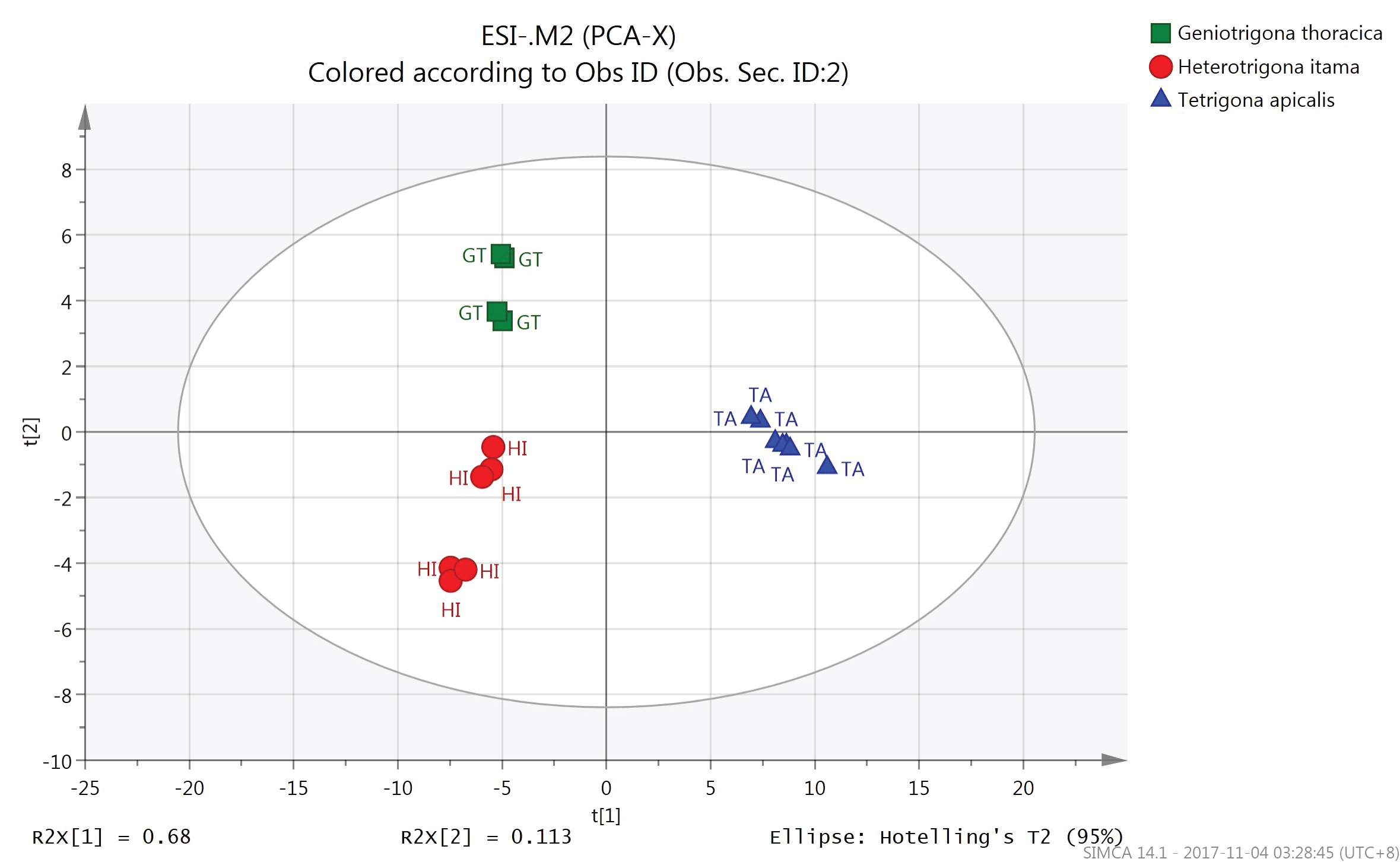

Supplement: Supplementary file 1 [file molecules-23-02160-s001.zip › Supplementary/Fig. 1-6c/Figure S4.tif]

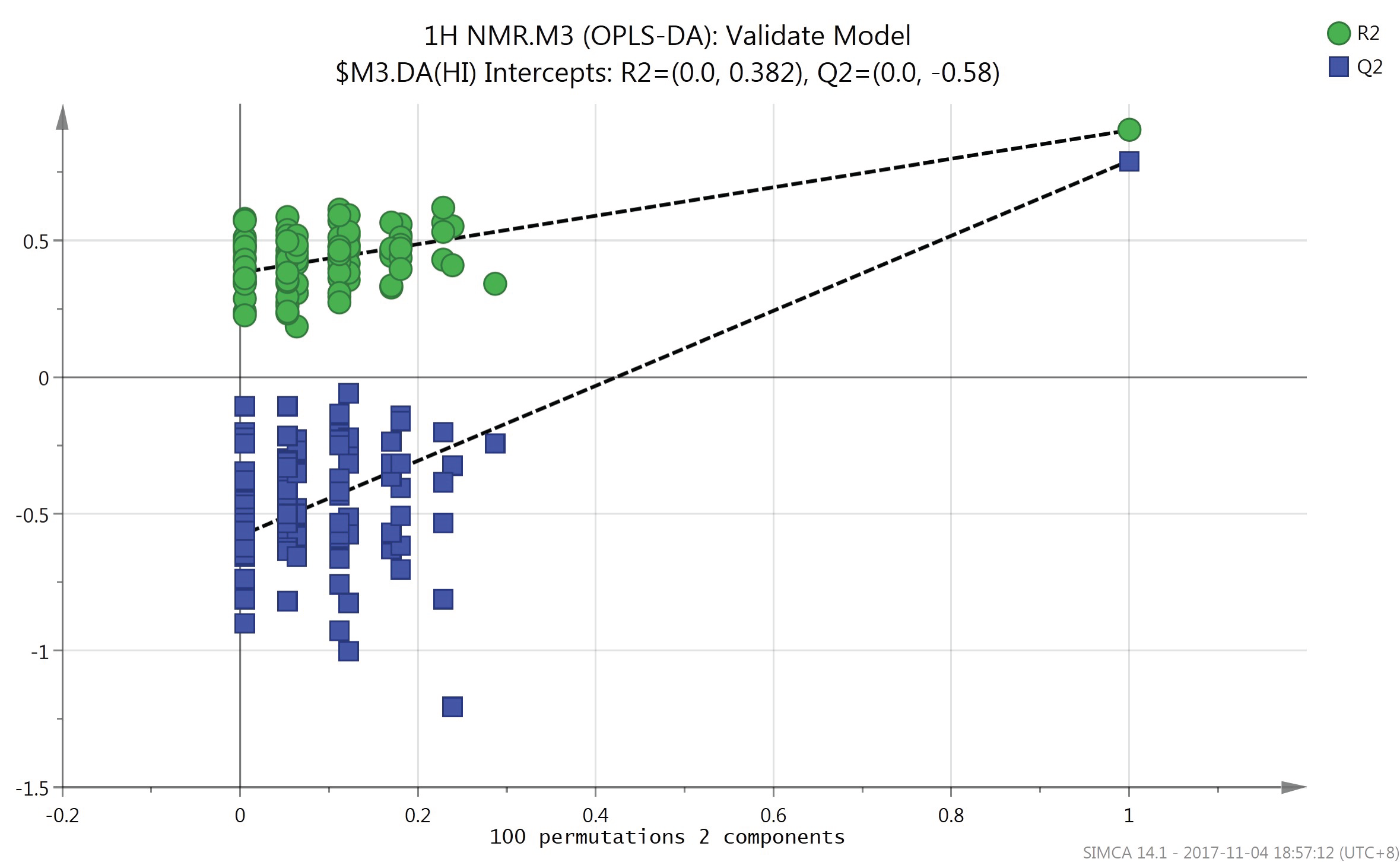

Supplement: Supplementary file 1 [file molecules-23-02160-s001.zip › Supplementary/Fig. 1-6c/Figure S5a.tif]

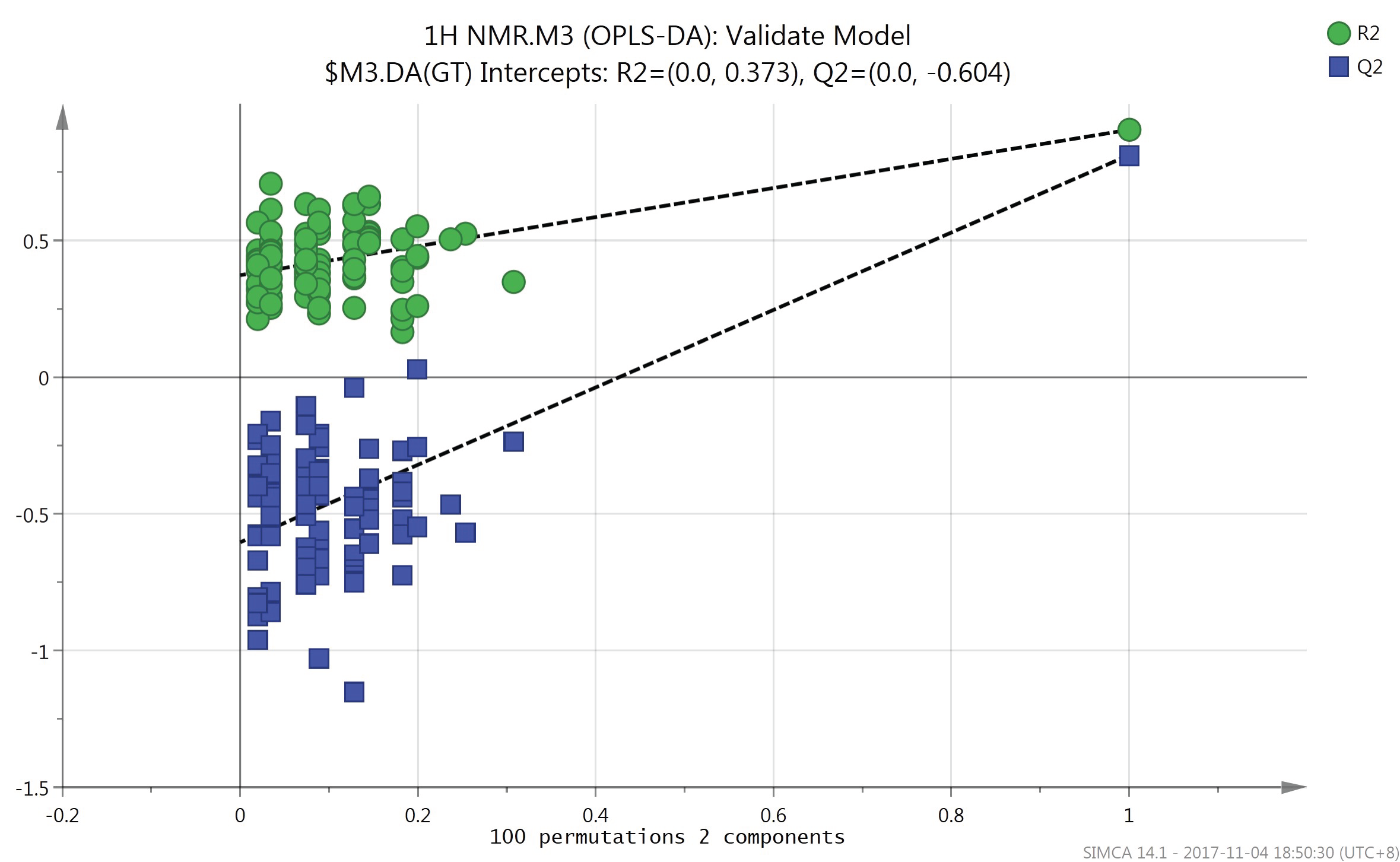

Supplement: Supplementary file 1 [file molecules-23-02160-s001.zip › Supplementary/Fig. 1-6c/Figure S5b.tif]

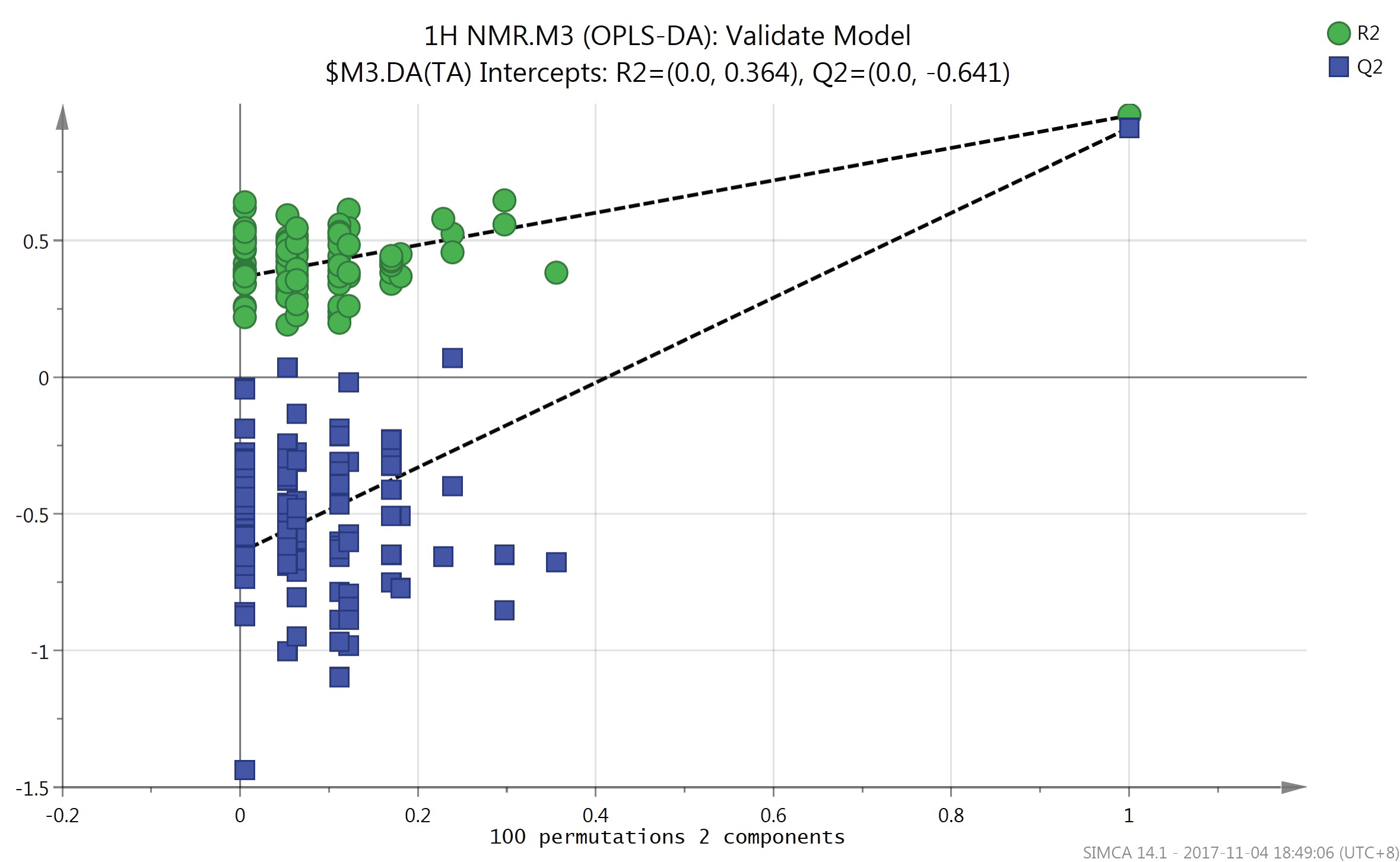

Supplement: Supplementary file 1 [file molecules-23-02160-s001.zip › Supplementary/Fig. 1-6c/Figure S5c.tif]

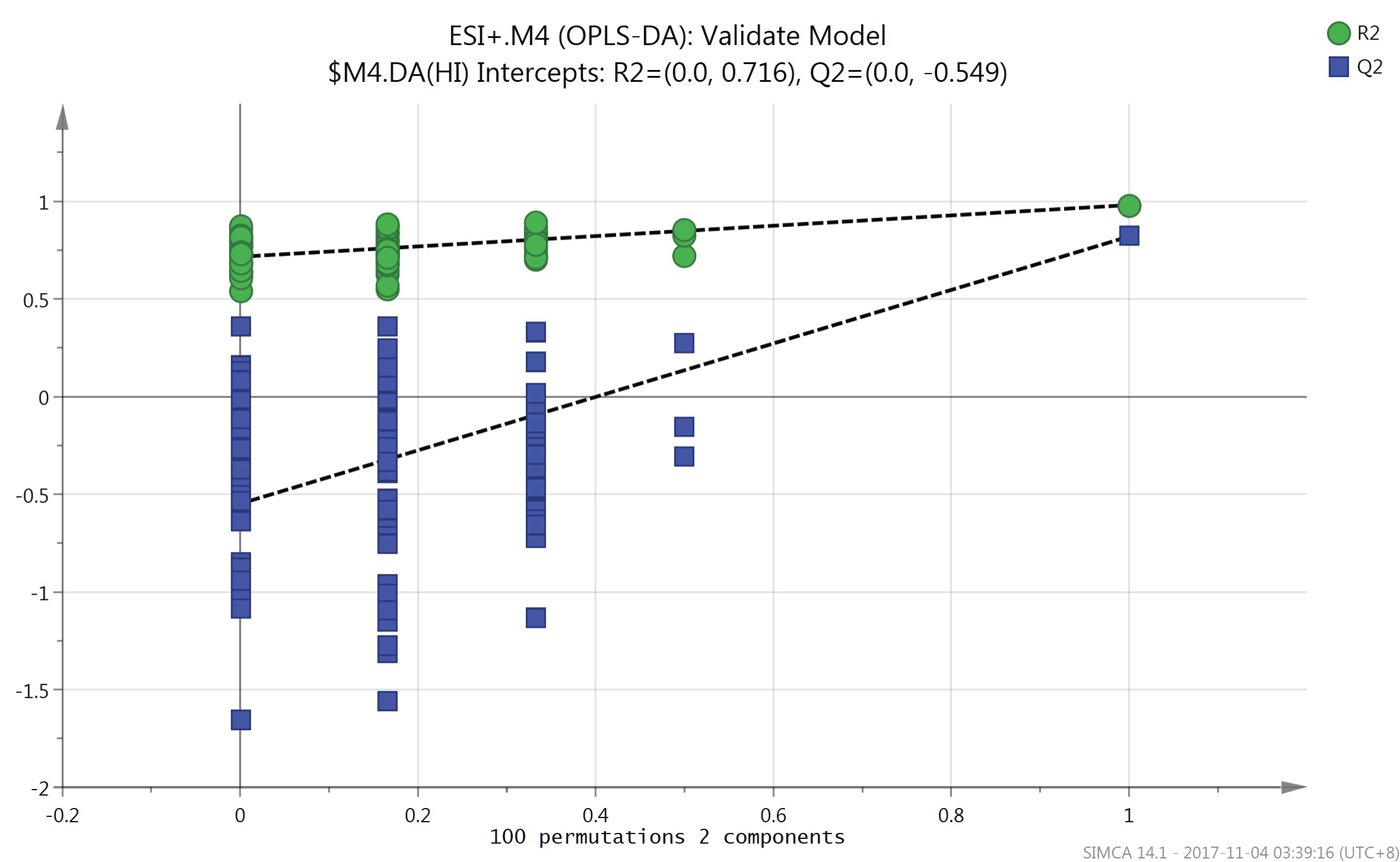

Supplement: Supplementary file 1 [file molecules-23-02160-s001.zip › Supplementary/Fig. 1-6c/Figure S6a.tif]

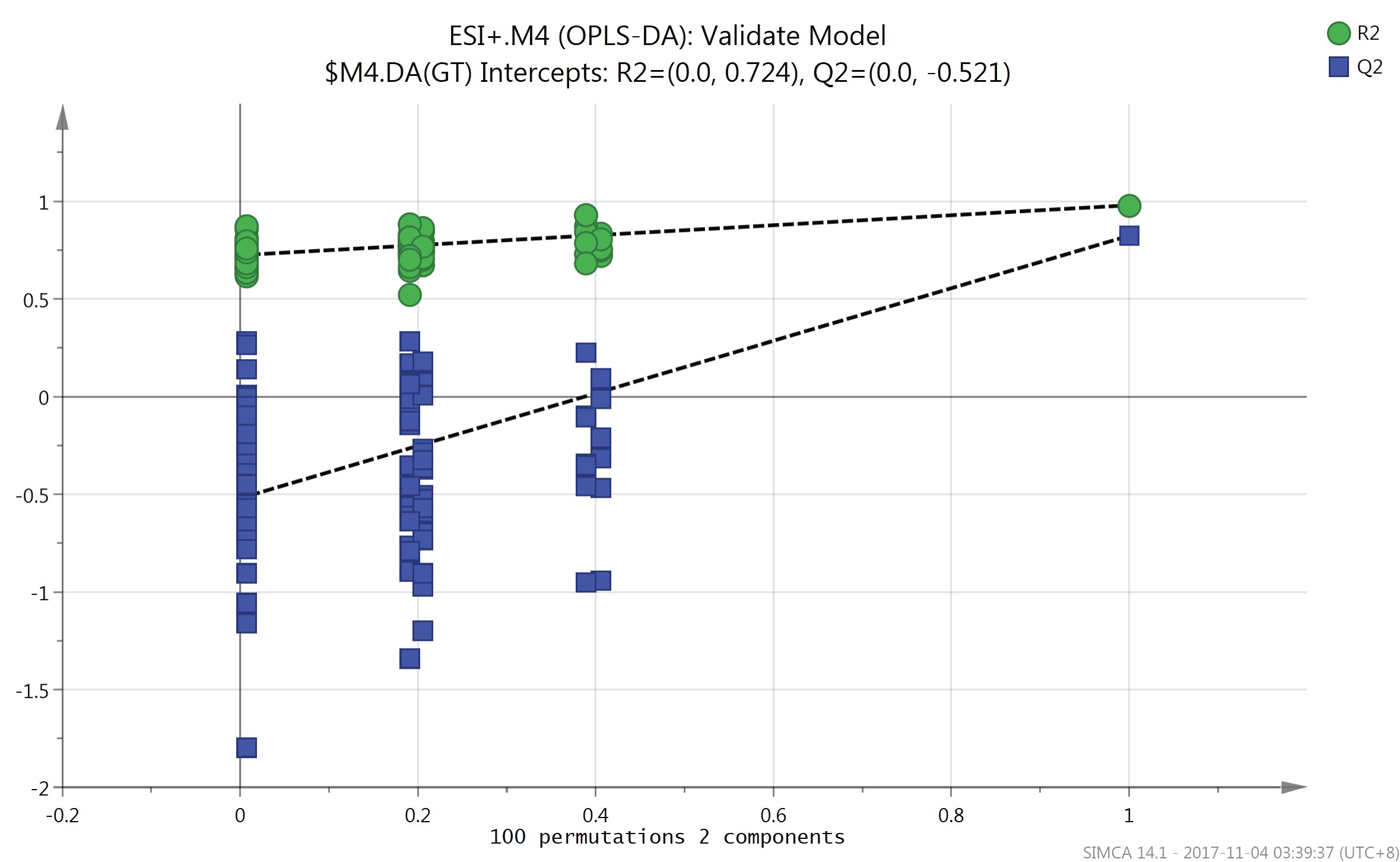

Supplement: Supplementary file 1 [file molecules-23-02160-s001.zip › Supplementary/Fig. 1-6c/Figure S6b.tif]

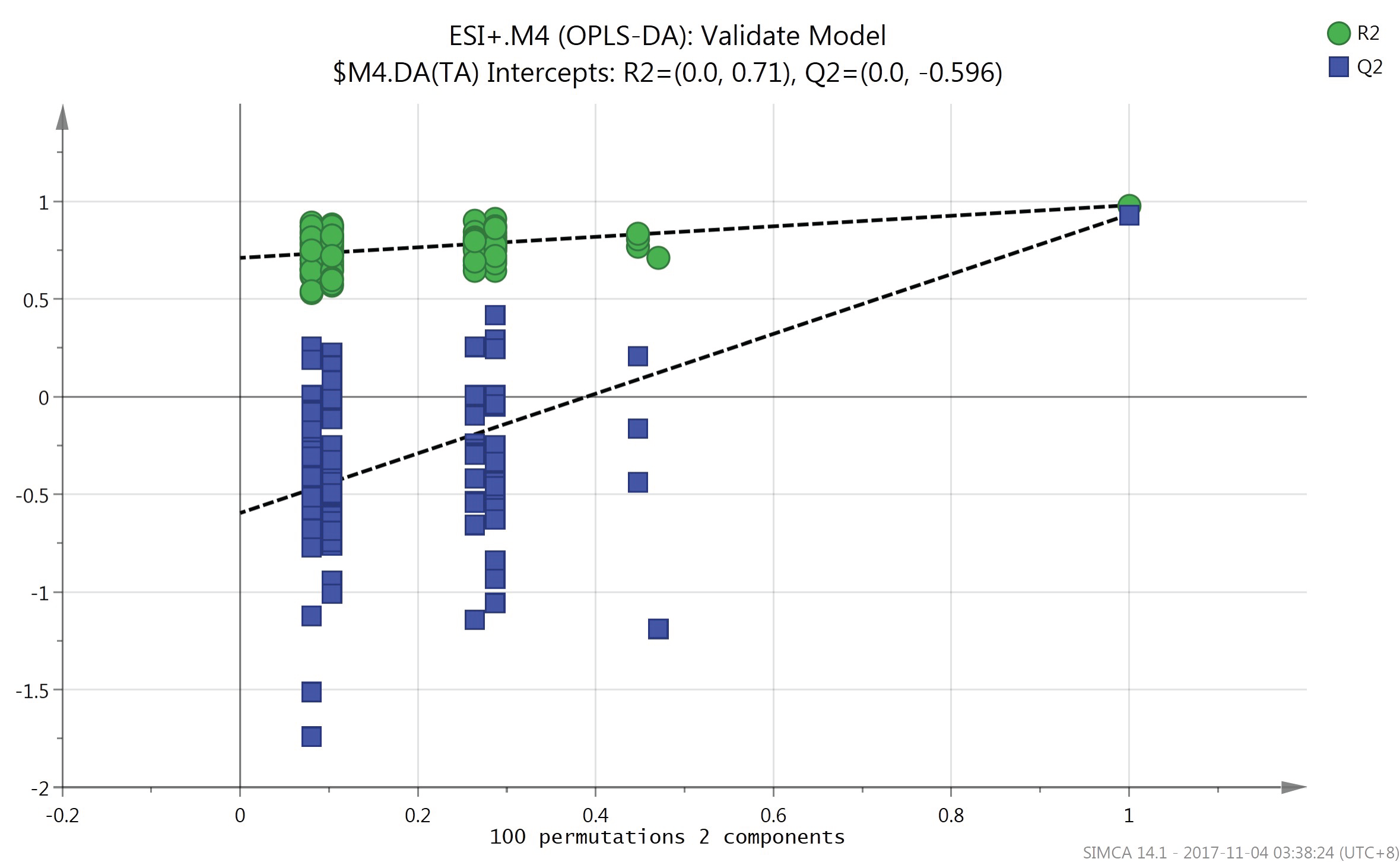

Supplement: Supplementary file 1 [file molecules-23-02160-s001.zip › Supplementary/Fig. 1-6c/Figure S6c.tif]

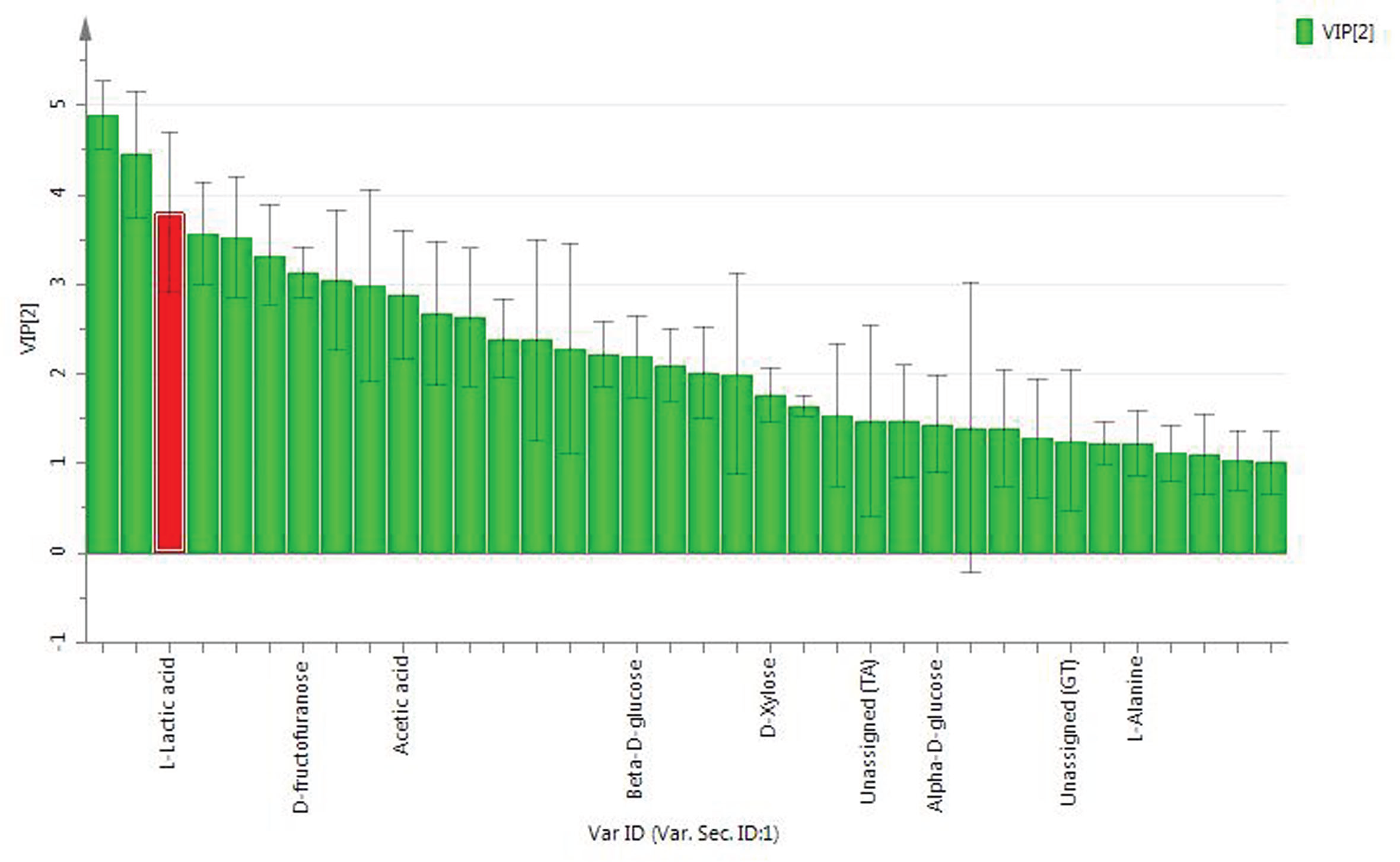

Supplement: Supplementary file 1 [file molecules-23-02160-s001.zip › Supplementary/Fig. 7a-10/Figure S10.tif]

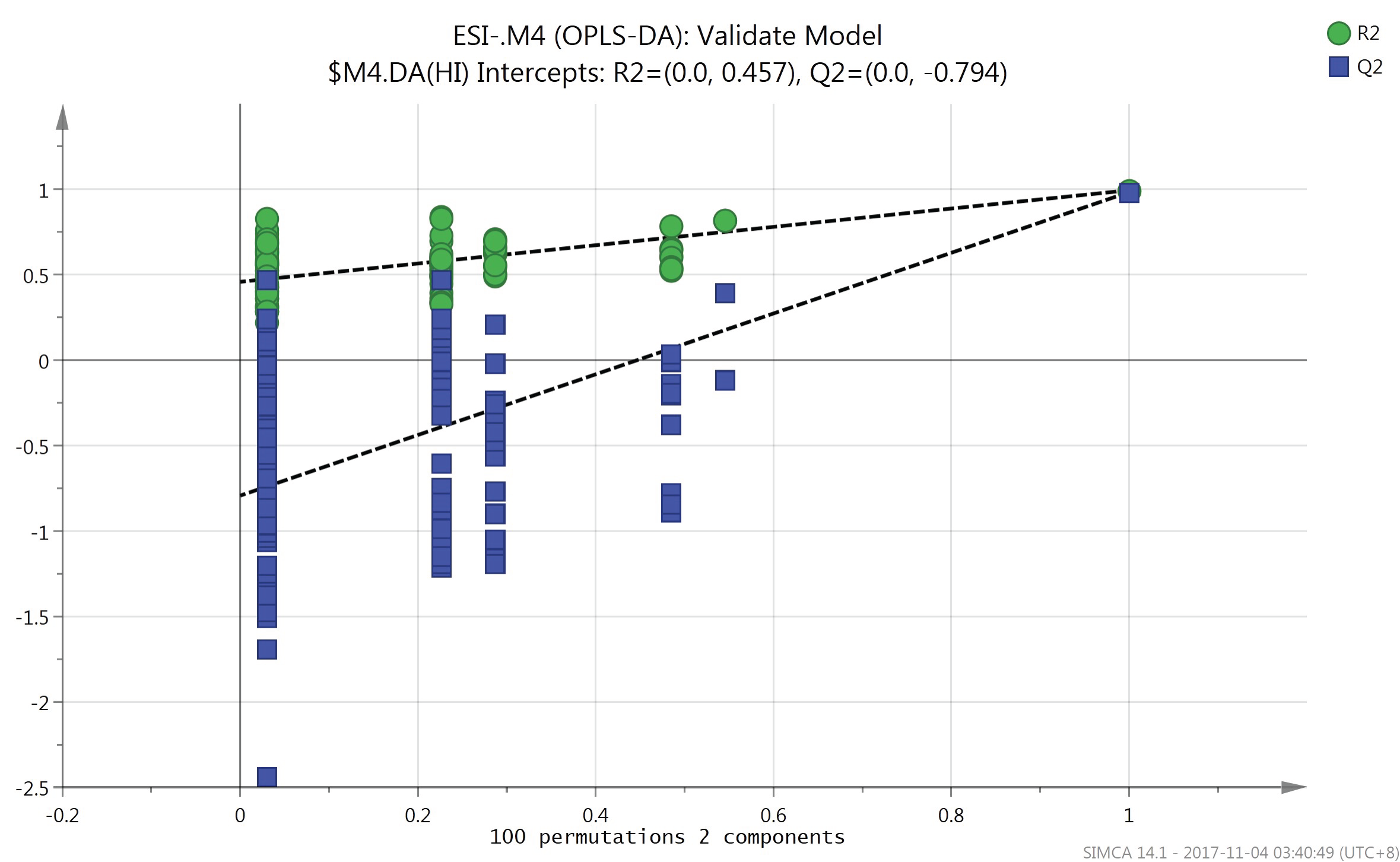

Supplement: Supplementary file 1 [file molecules-23-02160-s001.zip › Supplementary/Fig. 7a-10/Figure S7a.tif]

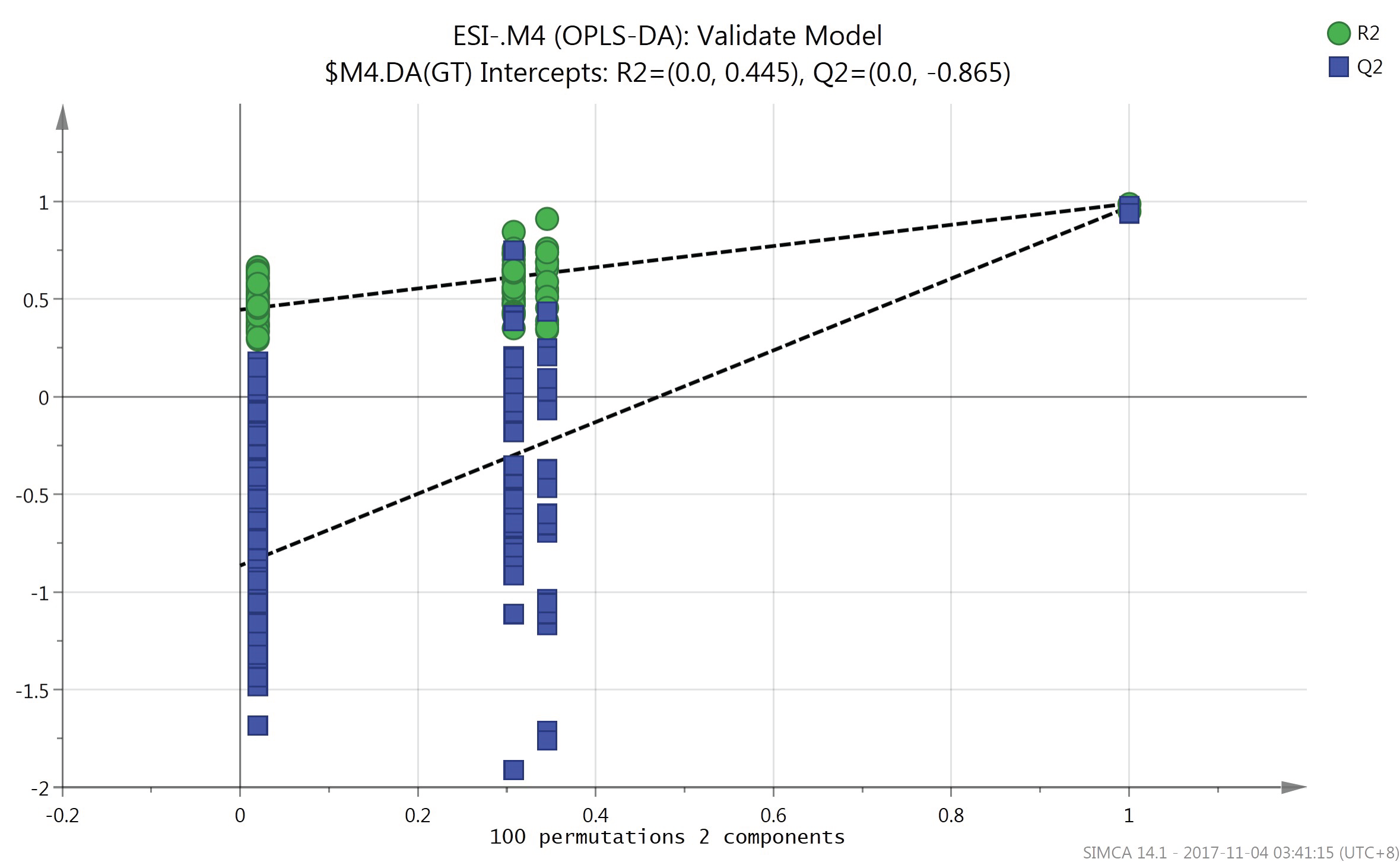

Supplement: Supplementary file 1 [file molecules-23-02160-s001.zip › Supplementary/Fig. 7a-10/Figure S7b.tif]

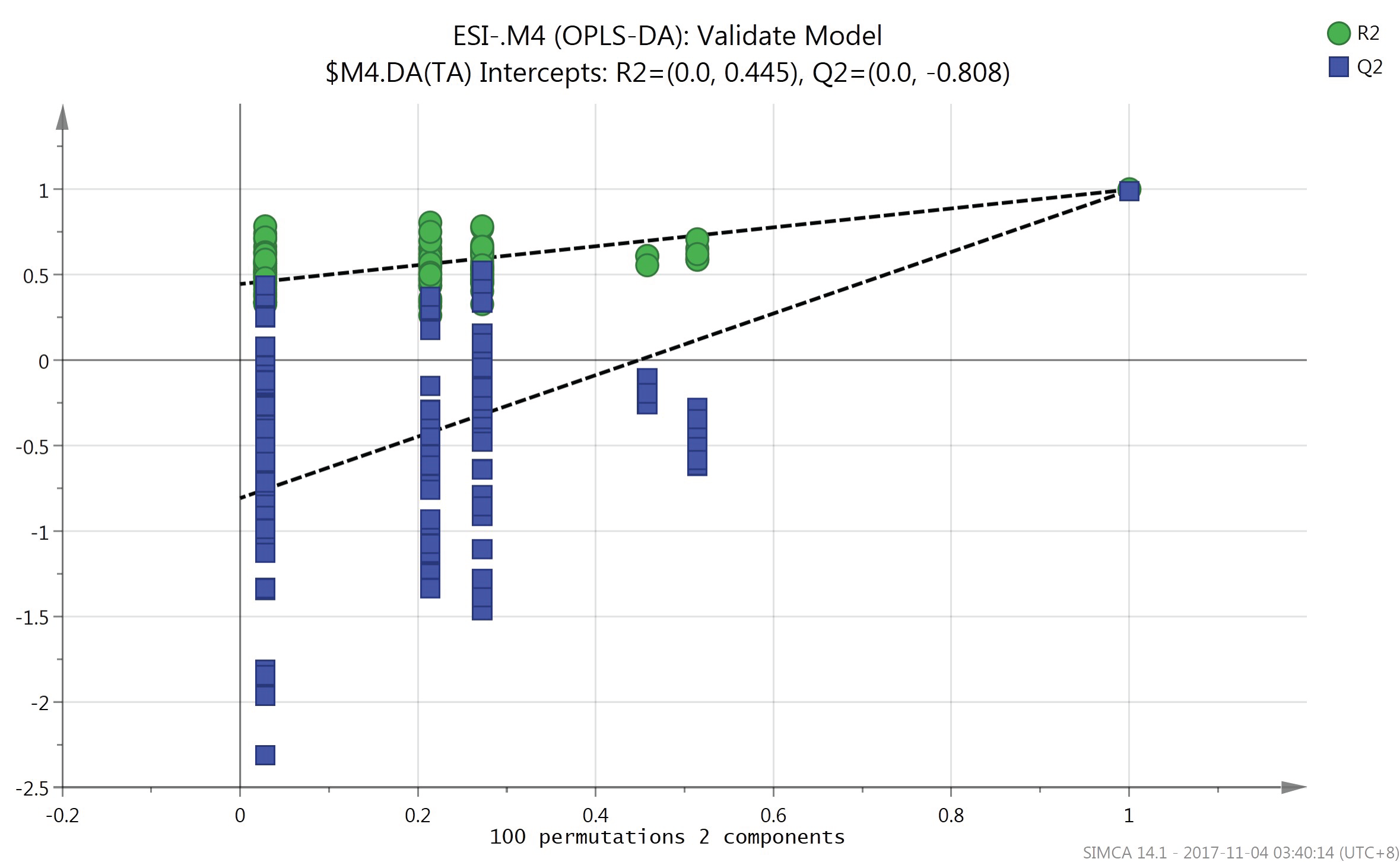

Supplement: Supplementary file 1 [file molecules-23-02160-s001.zip › Supplementary/Fig. 7a-10/Figure S7c.tif]

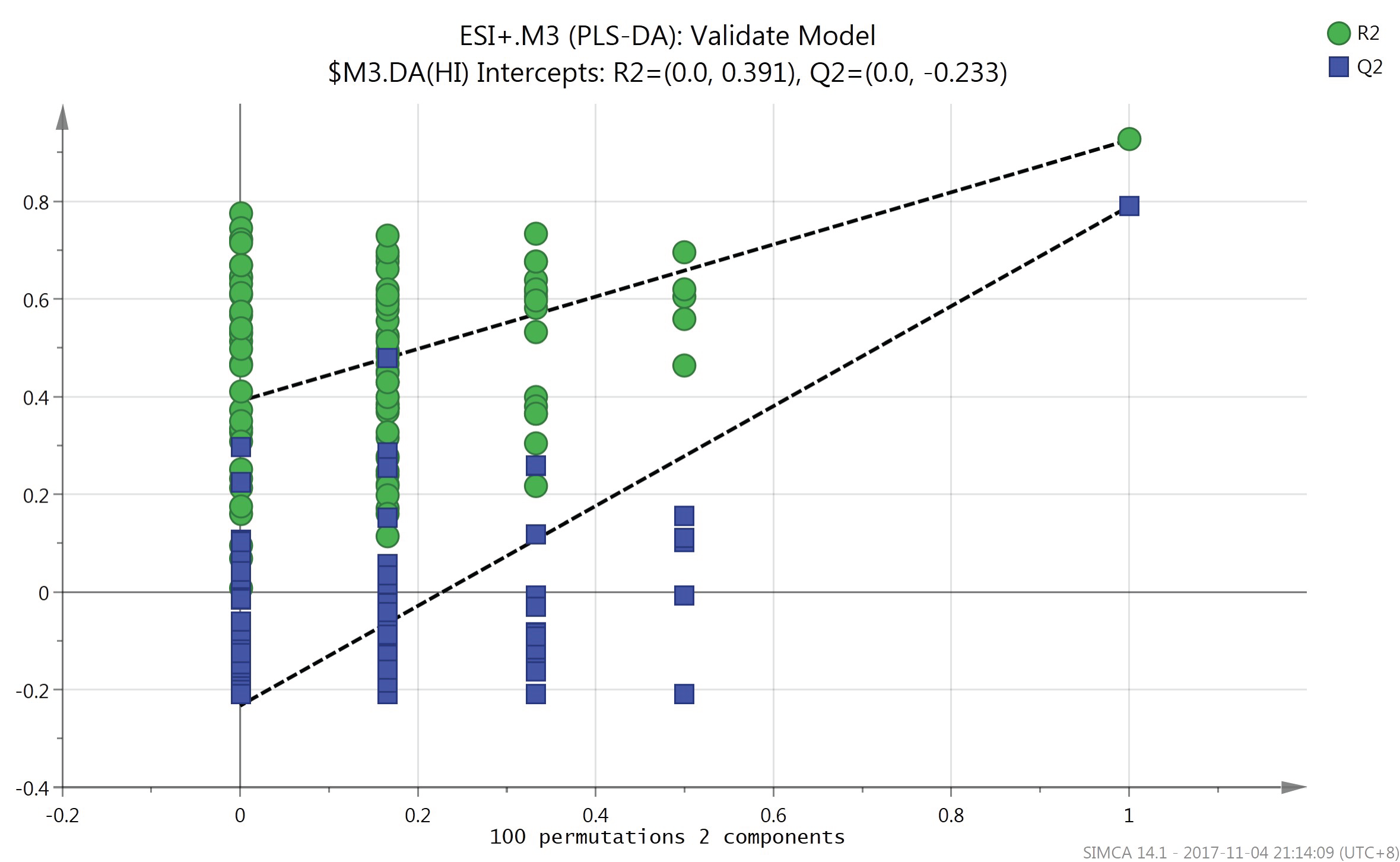

Supplement: Supplementary file 1 [file molecules-23-02160-s001.zip › Supplementary/Fig. 7a-10/Figure S8a.tif]

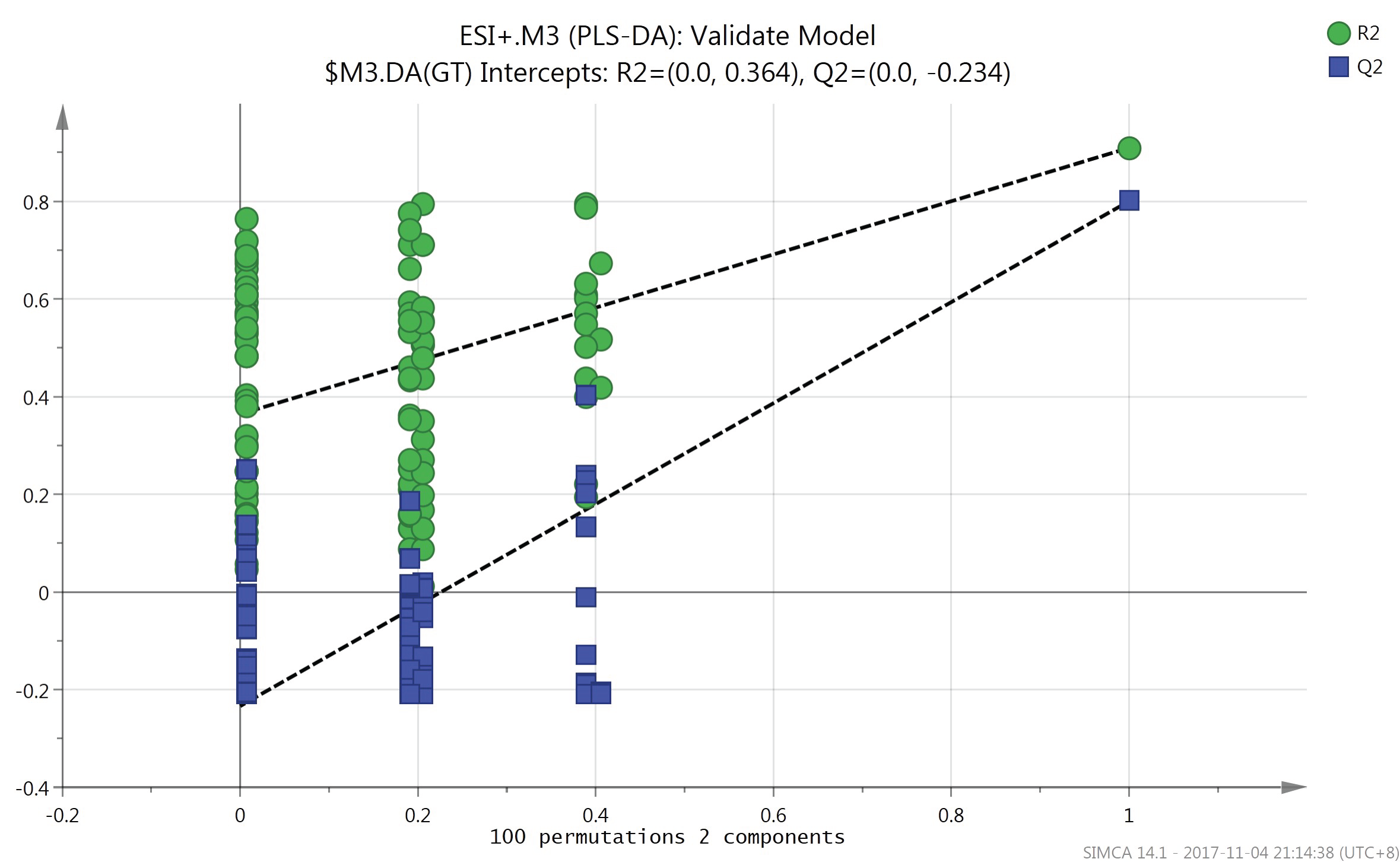

Supplement: Supplementary file 1 [file molecules-23-02160-s001.zip › Supplementary/Fig. 7a-10/Figure S8b.tif]

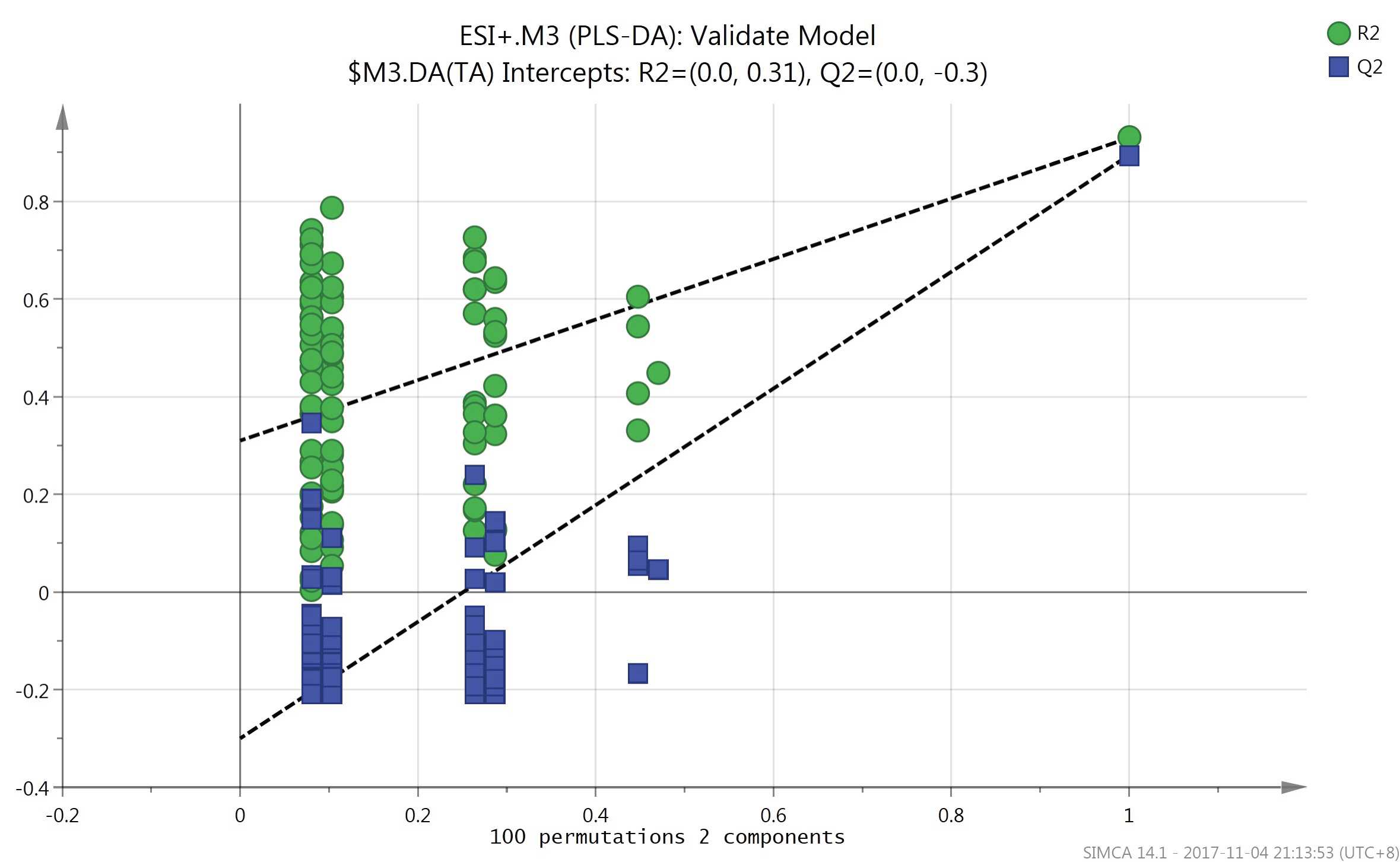

Supplement: Supplementary file 1 [file molecules-23-02160-s001.zip › Supplementary/Fig. 7a-10/Figure S8c.tif]

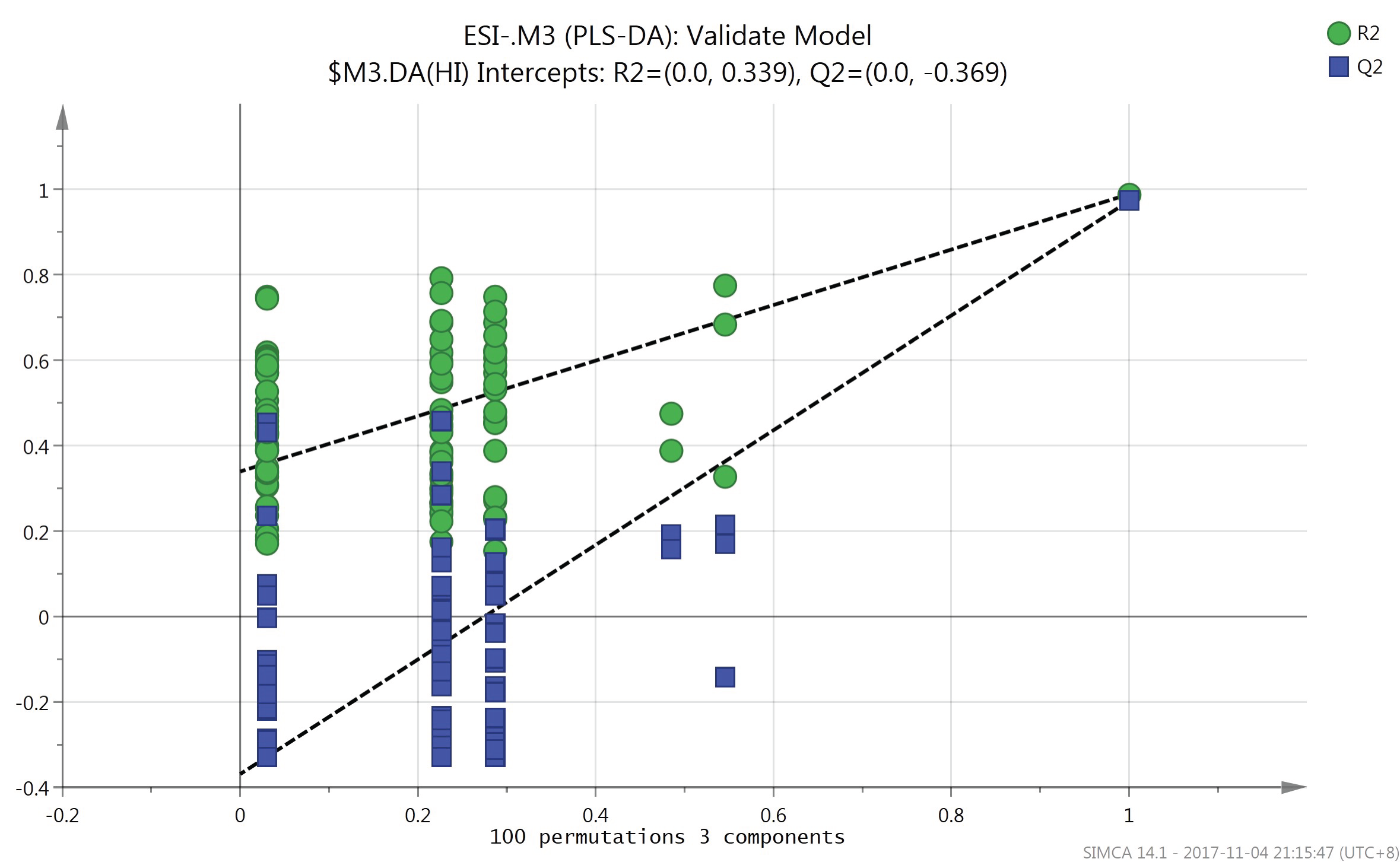

Supplement: Supplementary file 1 [file molecules-23-02160-s001.zip › Supplementary/Fig. 7a-10/Figure S9a.tif]

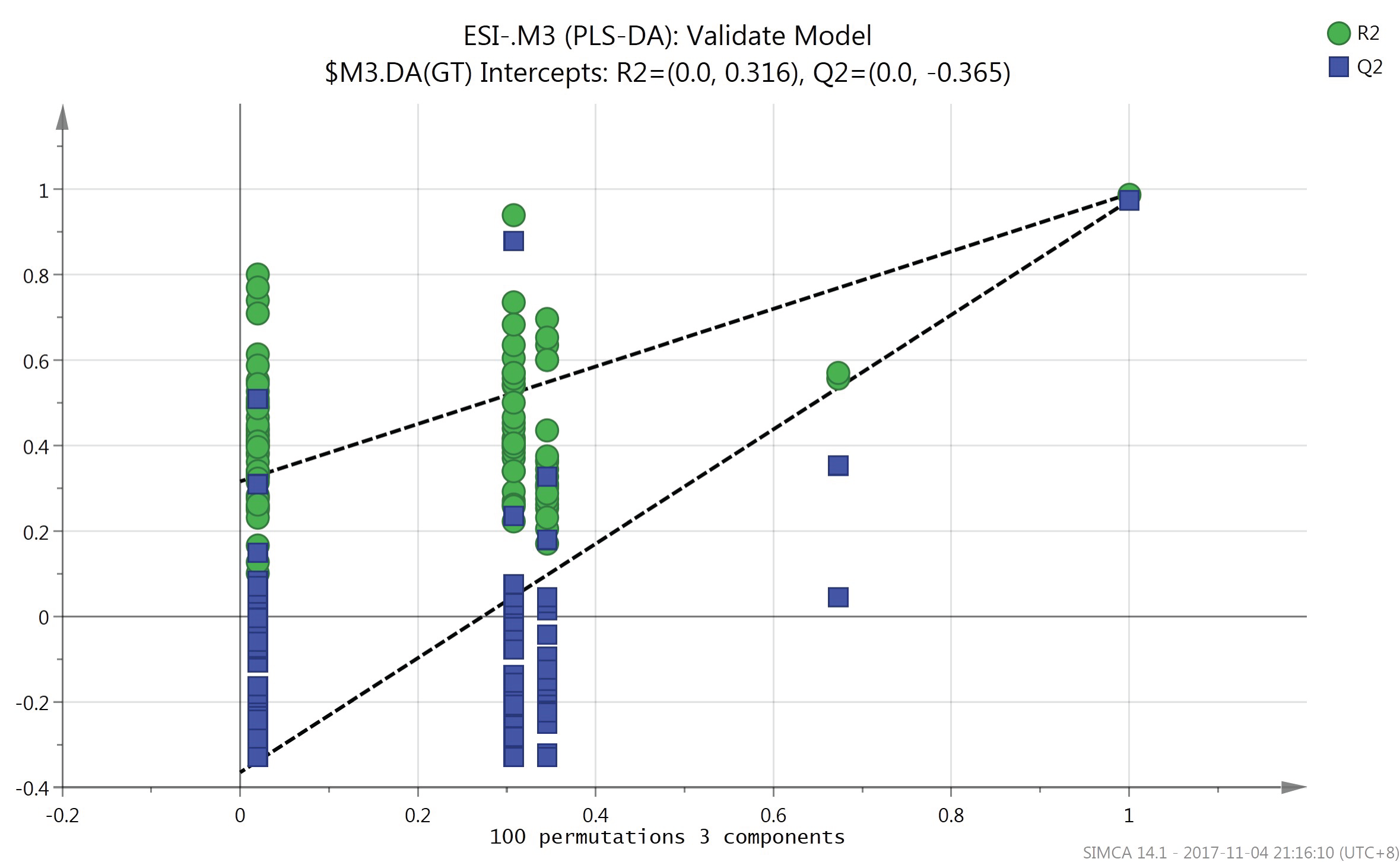

Supplement: Supplementary file 1 [file molecules-23-02160-s001.zip › Supplementary/Fig. 7a-10/Figure S9b.tif]

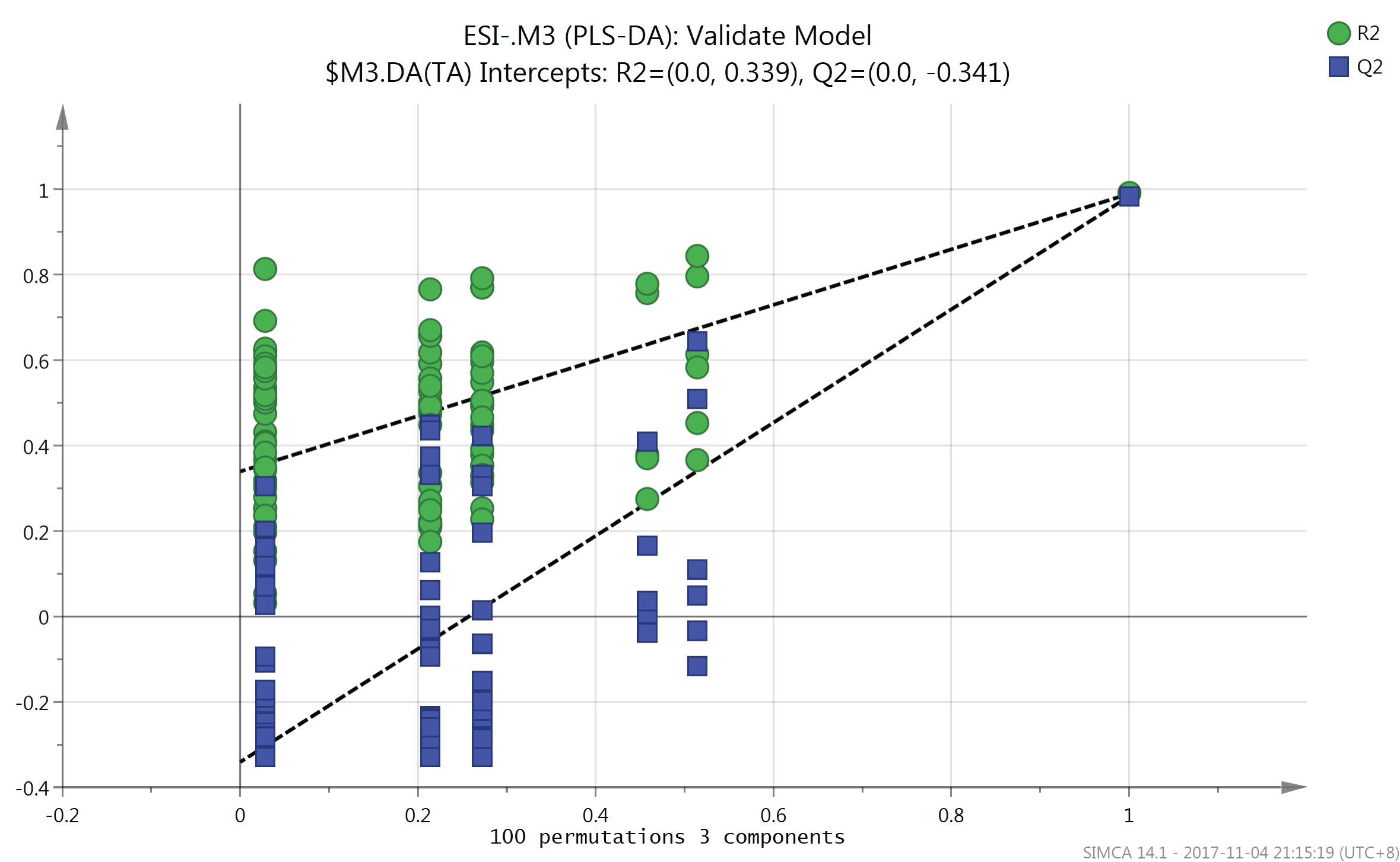

Supplement: Supplementary file 1 [file molecules-23-02160-s001.zip › Supplementary/Fig. 7a-10/Figure S9c.tif]

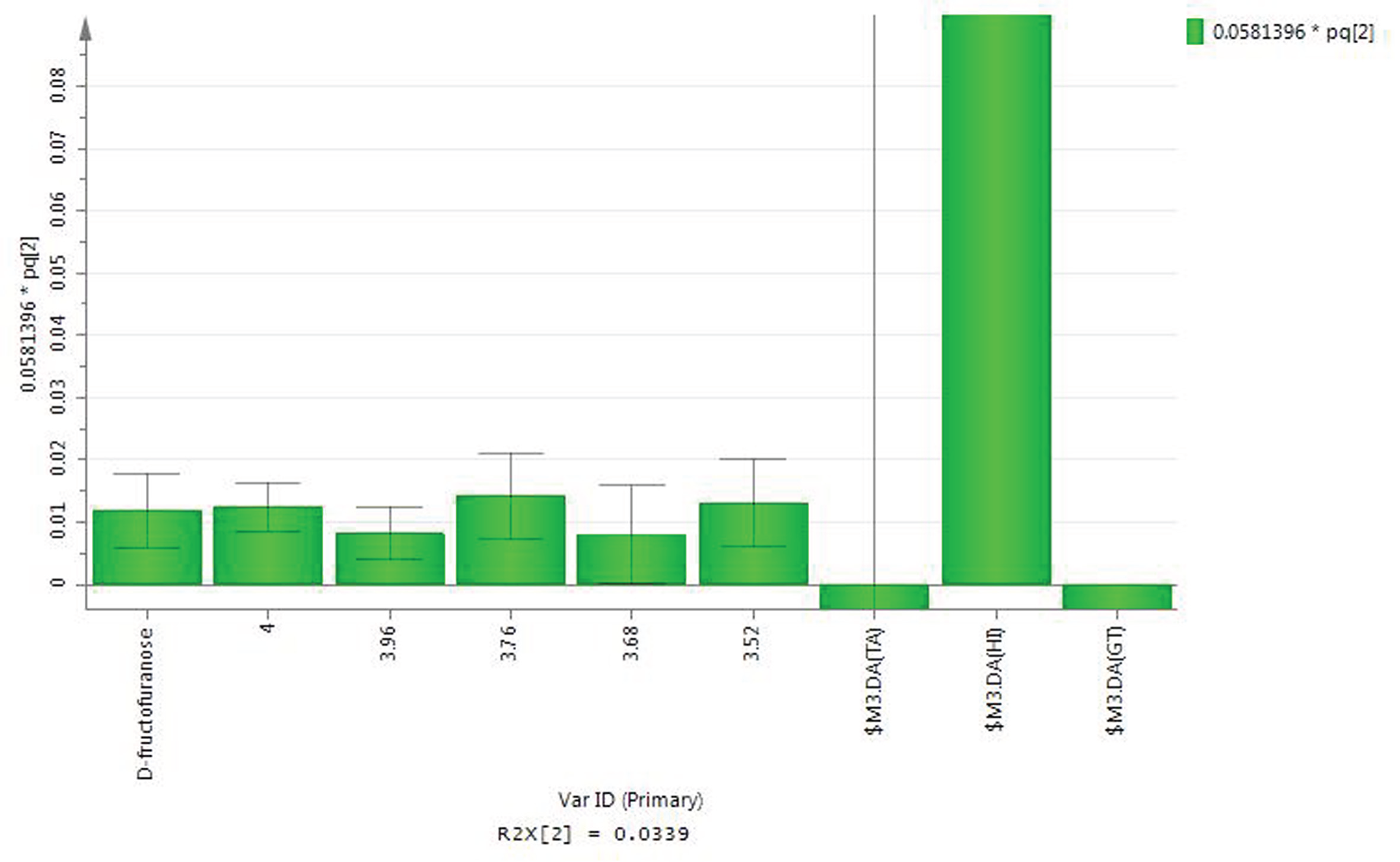

Supplement: Supplementary file 1 [file molecules-23-02160-s001.zip › Supplementary/Fig. S11a-S12a/Figure S11a.tif]

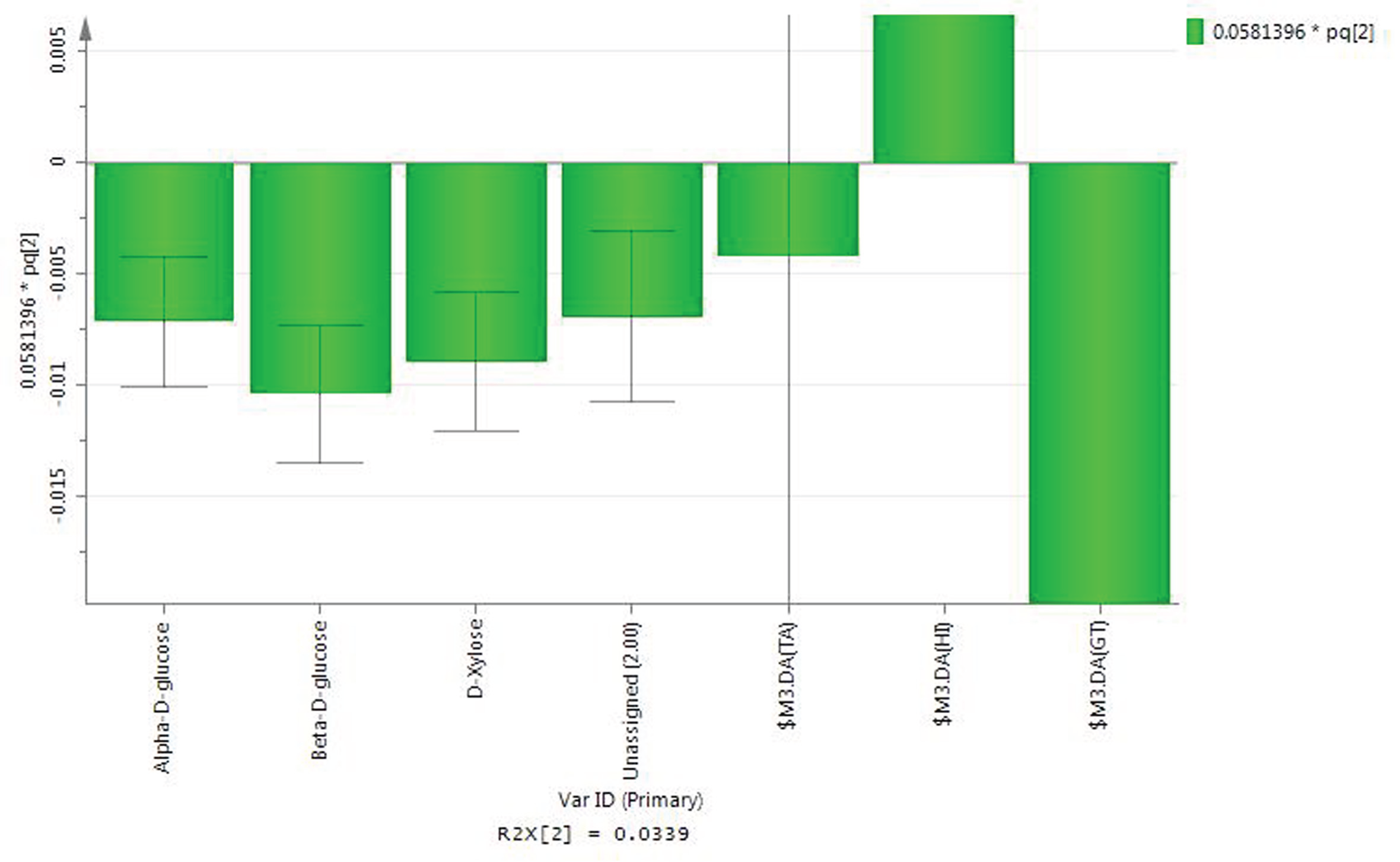

Supplement: Supplementary file 1 [file molecules-23-02160-s001.zip › Supplementary/Fig. S11a-S12a/Figure S11b.tif]

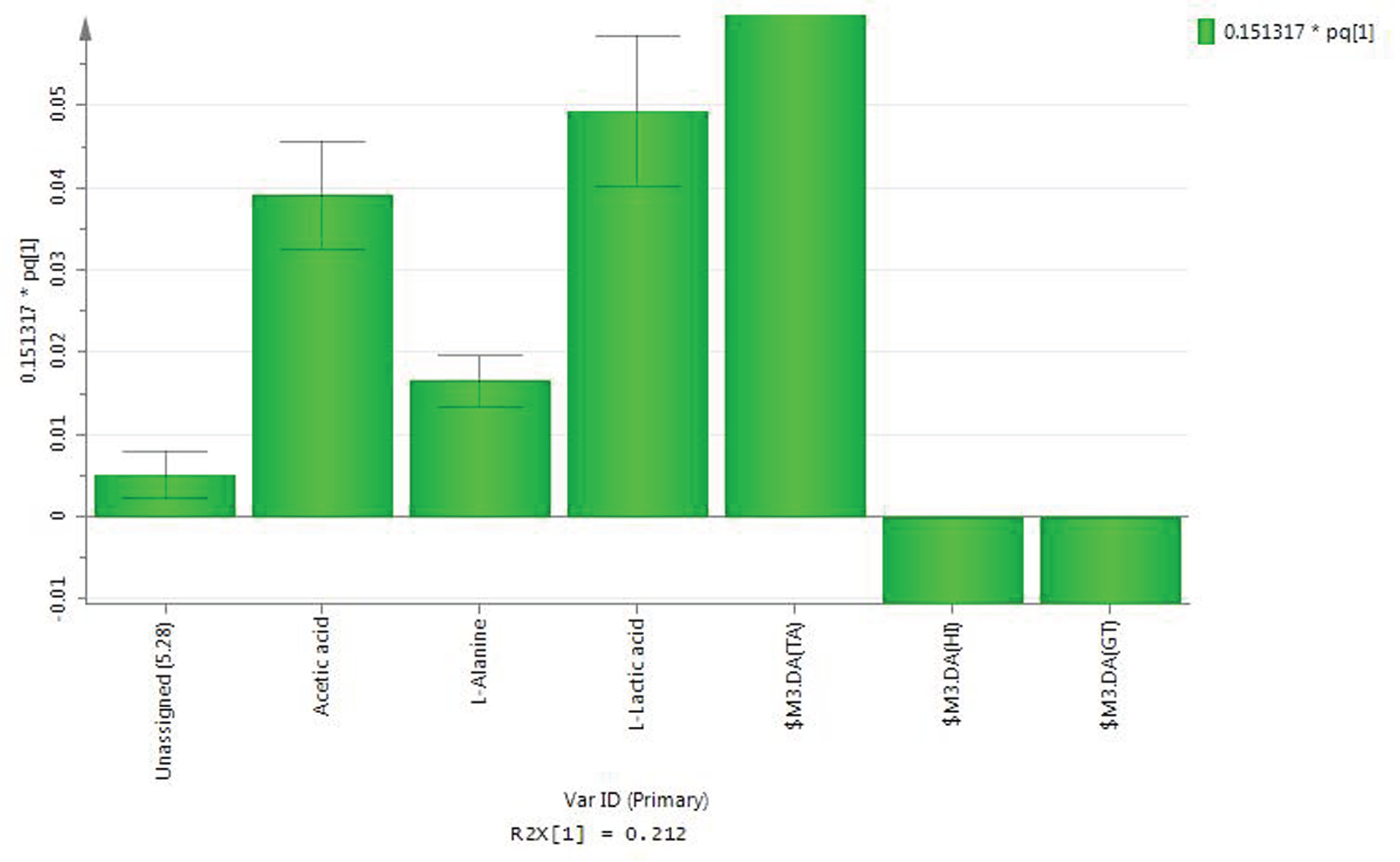

Supplement: Supplementary file 1 [file molecules-23-02160-s001.zip › Supplementary/Fig. S11a-S12a/Figure S11c.tif]

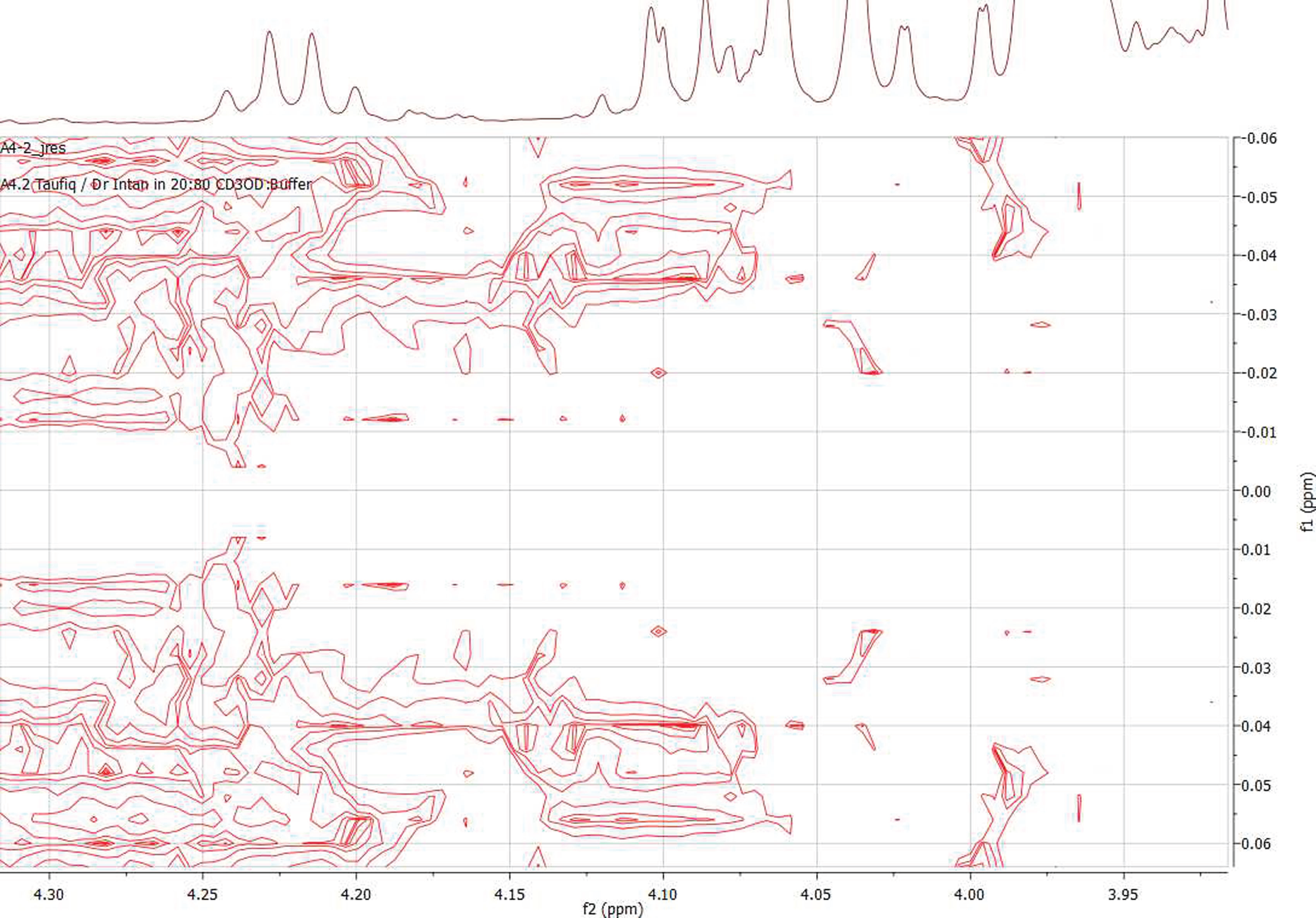

Supplement: Supplementary file 1 [file molecules-23-02160-s001.zip › Supplementary/Fig. S11a-S12a/Figure S12a.tif]

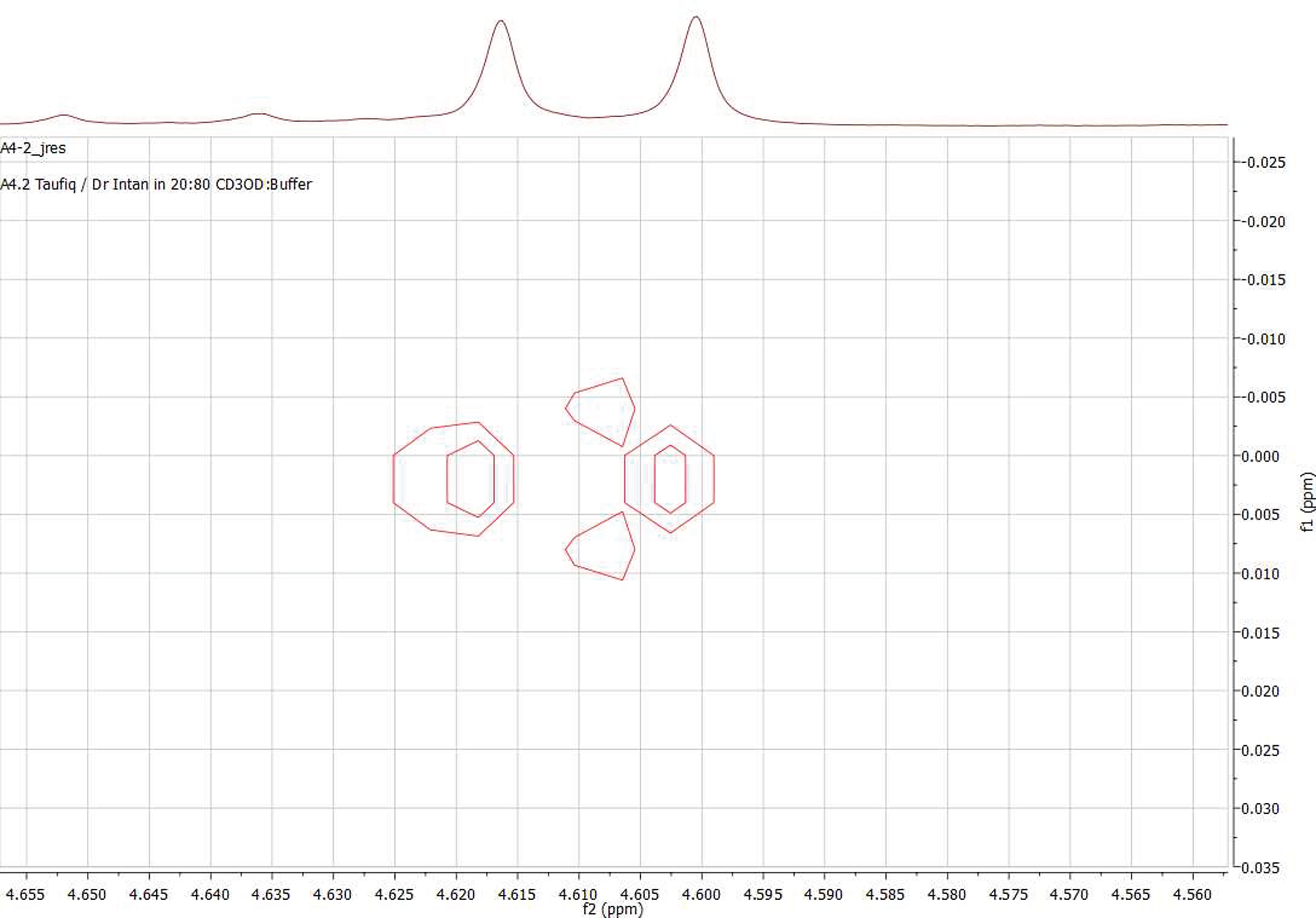

Supplement: Supplementary file 1 [file molecules-23-02160-s001.zip › Supplementary/Fig. S12b-S12c/Figure S12b.tif]

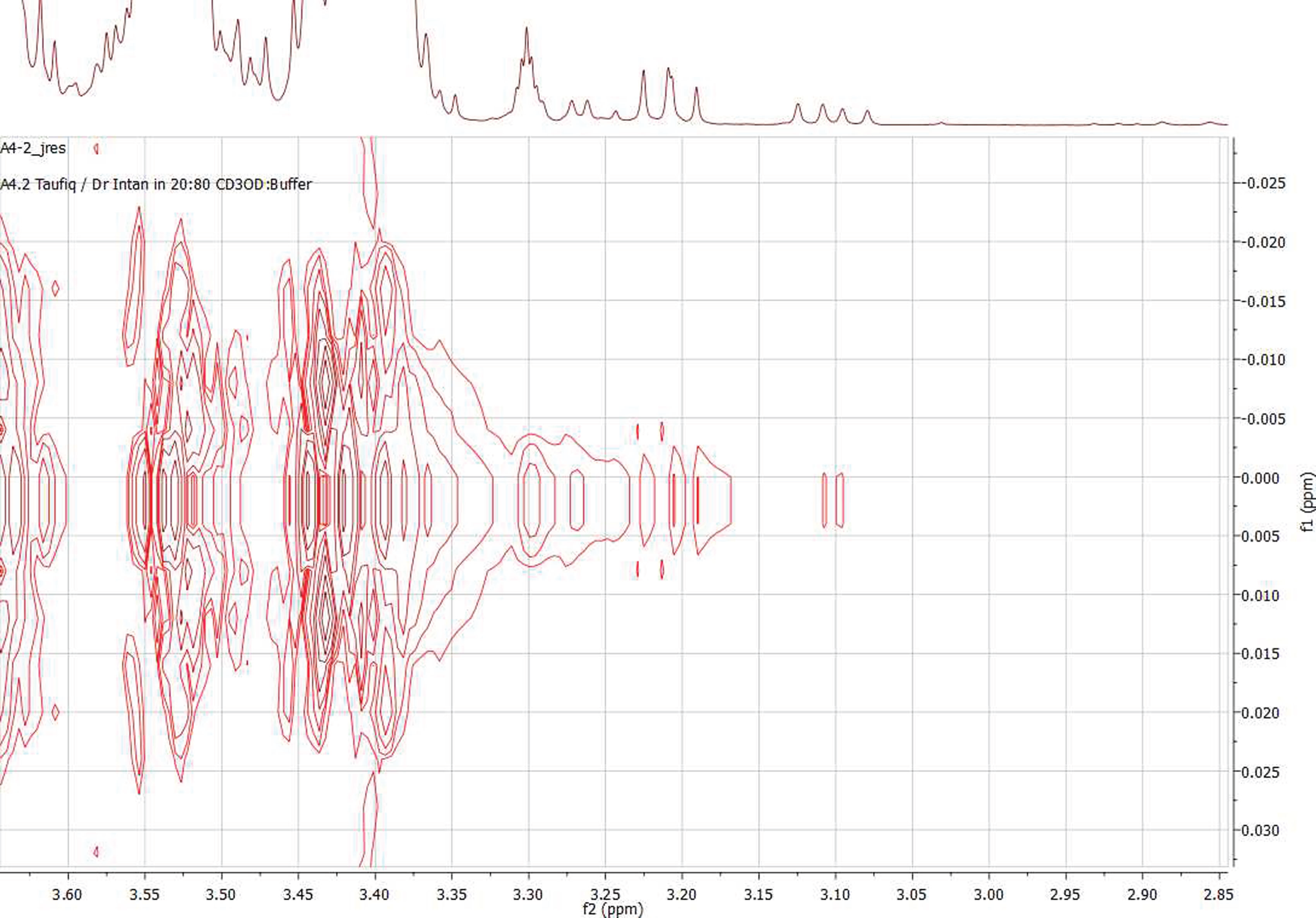

Supplement: Supplementary file 1 [file molecules-23-02160-s001.zip › Supplementary/Fig. S12b-S12c/Figure S12c.tif]

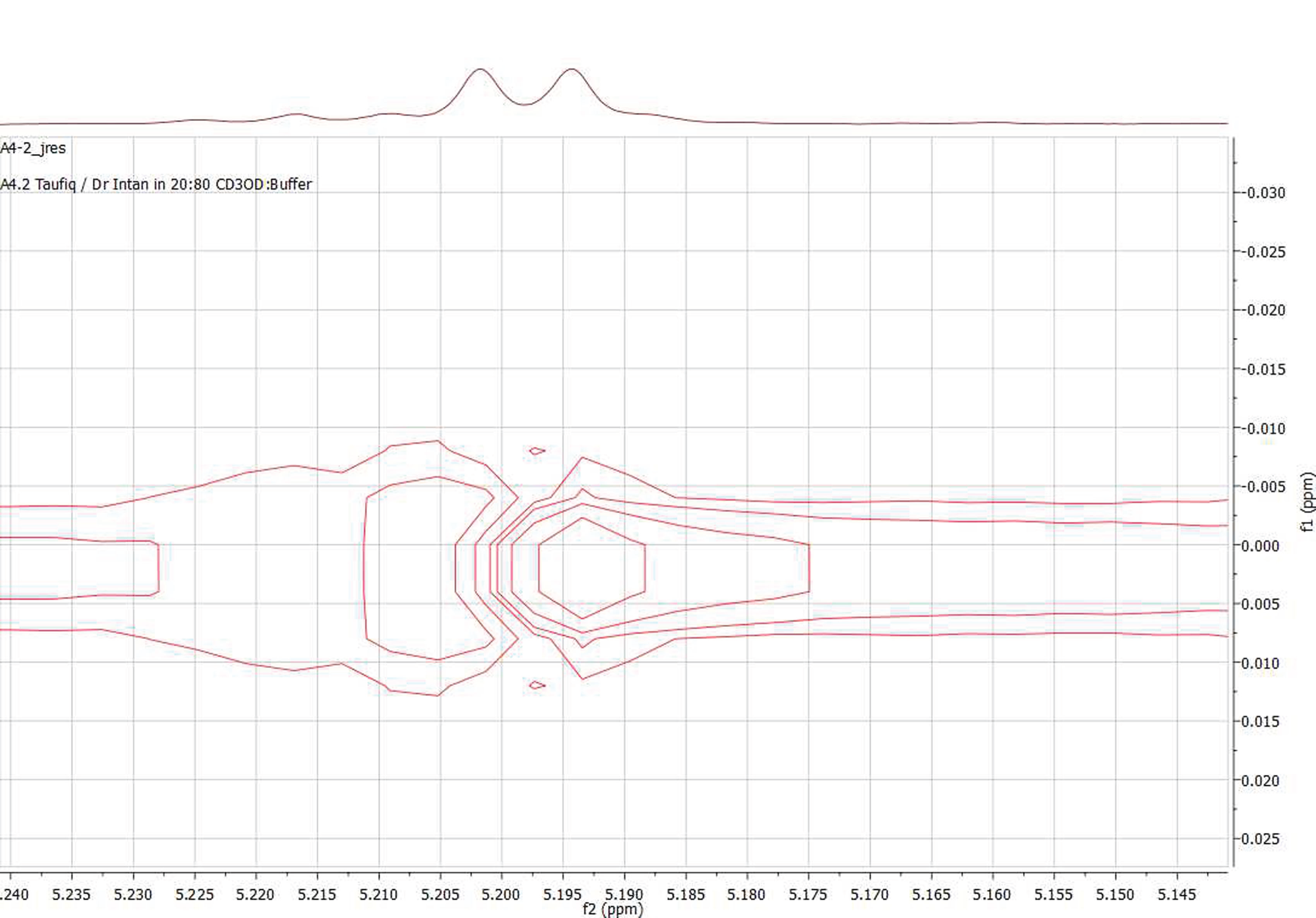

Supplement: Supplementary file 1 [file molecules-23-02160-s001.zip › Supplementary/Fig. S12d-S12e/Figure S12d.tif]

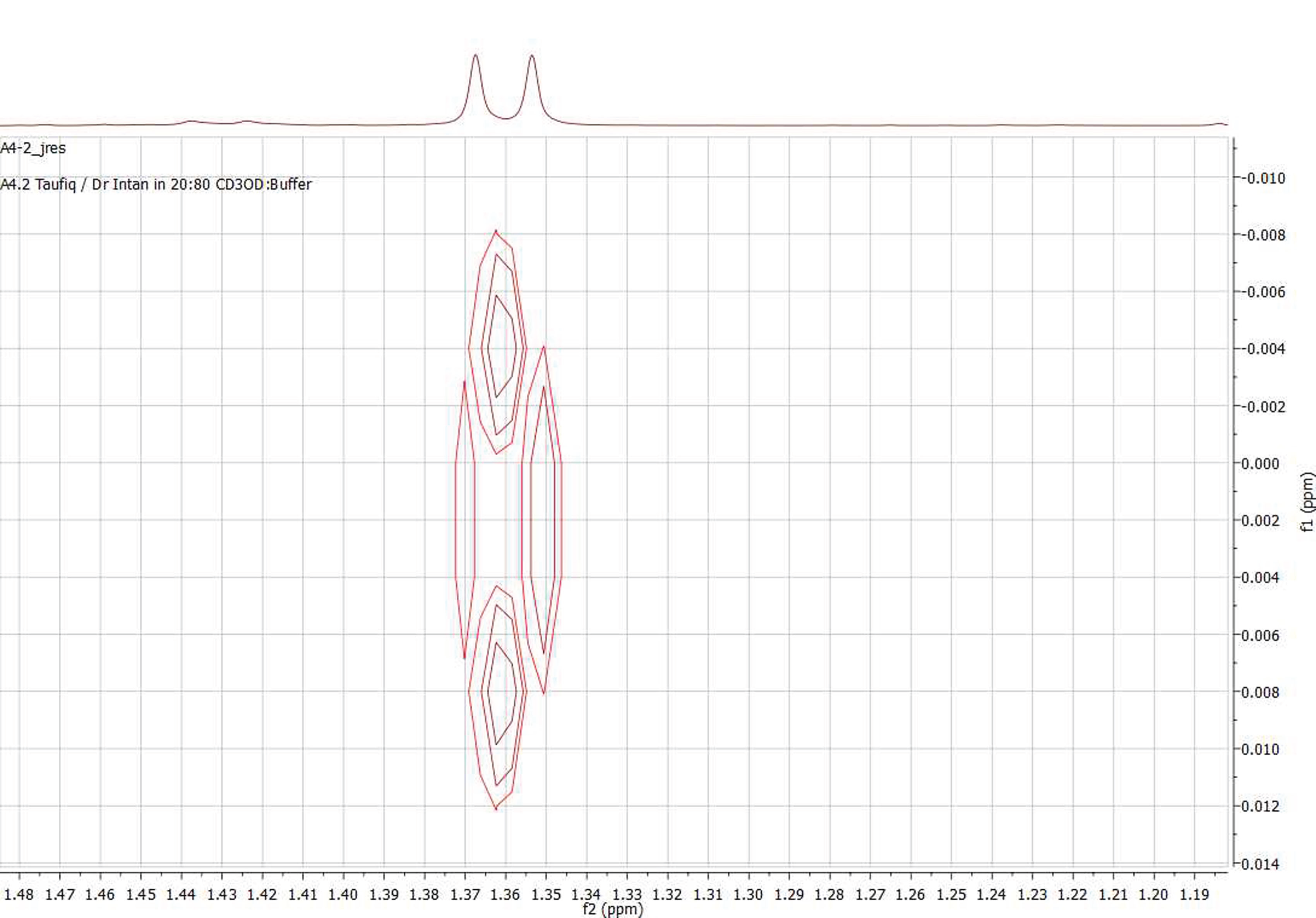

Supplement: Supplementary file 1 [file molecules-23-02160-s001.zip › Supplementary/Fig. S12d-S12e/Figure S12e.tif]

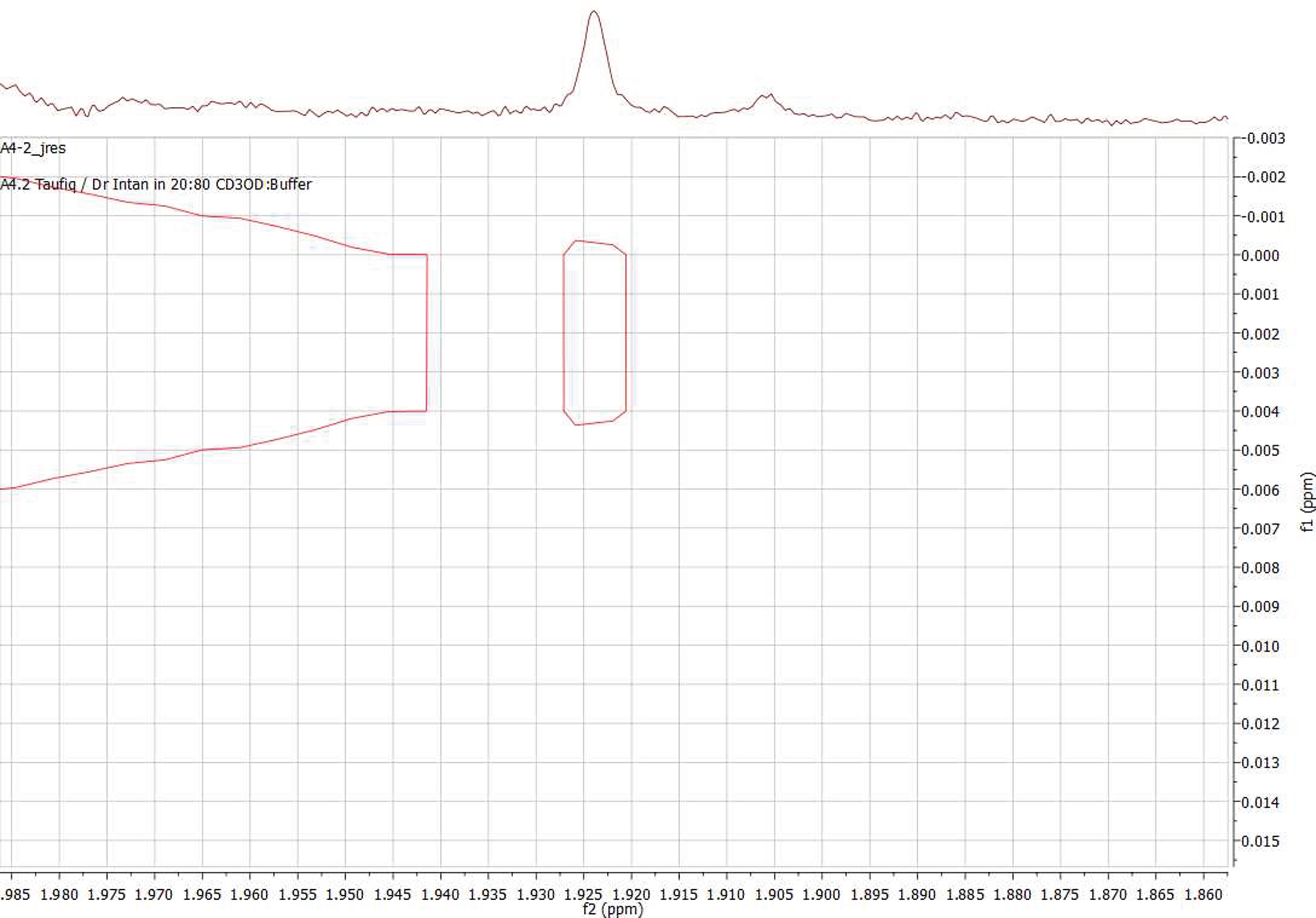

Supplement: Supplementary file 1 [file molecules-23-02160-s001.zip › Supplementary/Fig. S12f-S12g/Figure S12f.tif]

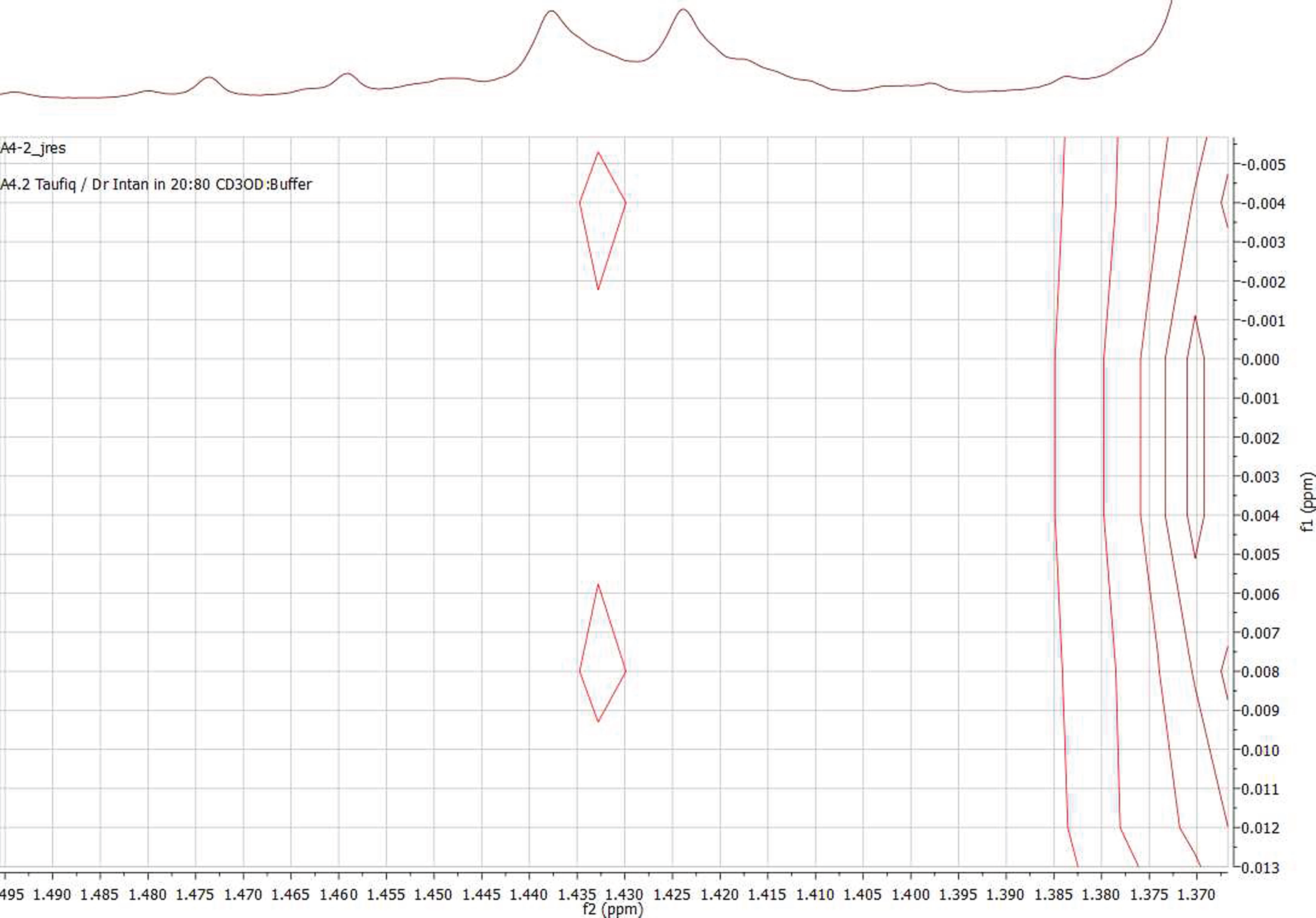

Supplement: Supplementary file 1 [file molecules-23-02160-s001.zip › Supplementary/Fig. S12f-S12g/Figure S12g.tif]
